# Synthetic Access to Aromatic α-Haloketones

**DOI:** 10.3390/molecules27113583

**Published:** 2022-06-02

**Authors:** Marre Porré, Gianmarco Pisanò, Fady Nahra, Catherine S. J. Cazin

**Affiliations:** 1 Department of Chemistry and Center for Sustainable Chemistry, Ghent University, 9000 Ghent, Belgium; marreporre@outlook.be (M.P.); gianmarco.pisano@ugent.be (G.P.); fady.nahra@vito.be (F.N.); 2 VITO (Flemish Institute for Technological Research), Separation and Conversion Technology, Boeretang 200, 2400 Mol, Belgium

**Keywords:** haloketones, building block, organic synthesis

## Abstract

α-Haloketones play an essential role in the synthesis of complex *N*-, *S*-, *O*-heterocycles; of which some exhibit a remarkable biological activity. Research further illustrated that α-bromo-, α-chloro-, and α-iodoketones are key precursors for blockbuster pharmacological compounds. Over the past twenty years, substantial advances have been made in the synthesis of these industrially relevant building blocks. Efforts have focused on rendering the synthetic protocols greener, more effective and versatile. In this survey, we summarised and thoroughly evaluated the progress of the field, established in the past two decades, in terms of generality, efficacy and sustainability.


**Table of Contents**

1. Introduction............................................................................................................................................................................................................22. Direct α-Halogenation of Aryl Ketones..............................................................................................................................................................3 2.1. Electrophilic Halogenation............................................................................................................................................................................3  2.1.1. Br_2_ as Halogenation Agent.....................................................................................................................................................................3  2.1.2. I_2_ as Halogenation Agent........................................................................................................................................................................6  2.1.3. NXS as Halogenation Agents.................................................................................................................................................................11  2.1.4. Halides (X^−^) as X_2_ Sources......................................................................................................................................................................15   Metal Halides (MX).......................................................................................................................................................................................15   Quaternary Ammonium Halides (NH4X)...................................................................................................................................................16   Hydrogen Halides (HX)................................................................................................................................................................................17  2.1.5. Polymeric Halogenation Agents............................................................................................................................................................20  2.1.6. Ionic Liquids as Halogenation Agent....................................................................................................................................................22  2.1.7. Miscellaneous Halogenation Agents.....................................................................................................................................................24 2.2. Nucleophilic Halogenation...........................................................................................................................................................................263. Oxyhalogenation of Hydrocarbons.....................................................................................................................................................................28 3.1. Alkenes as Substrates.....................................................................................................................................................................................28 3.2. Alkynes as Substrates.....................................................................................................................................................................................324. Oxidative Halogenation of Secondary Alcohols................................................................................................................................................345. Oxidation of Functionalised Hydrocarbons.......................................................................................................................................................35 5.1. Haloalkanes as Substrates.............................................................................................................................................................................35 5.2. Haloalkenes as Substrates.............................................................................................................................................................................36 5.3. Haloalcohols as Substrates............................................................................................................................................................................376. Hydration of 1-Haloalkynes................................................................................................................................................................................38 6.1. Metal-Catalysed Hydrations.........................................................................................................................................................................38 6.2. Non-Metal-Catalysed Hydrations................................................................................................................................................................407. Miscellaneous Routes...........................................................................................................................................................................................41 7.1. α-Functionalised Ketones as Substrates......................................................................................................................................................41 7.2. Alternative Substrates....................................................................................................................................................................................438. Conclusions and Outlook.....................................................................................................................................................................................45References..................................................................................................................................................................................................................46

## 1. Introduction

**Figure 1:**
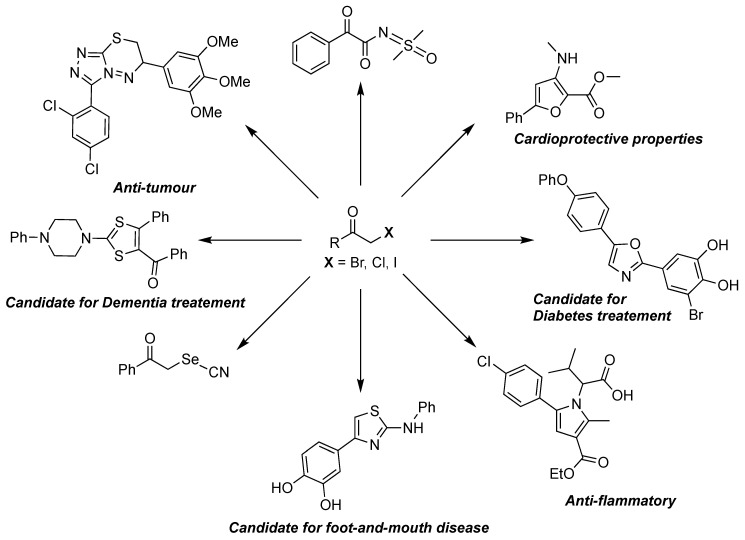
α-Haloketones as versatile building blocks in organic synthesis.

Over the past few decades, the versatility and synthetic value of α-haloketones in organic synthesis have been amply showcased in the literature. The existence of two adjacent electrophilic centres, namely the α-halocarbon and the carbonyl group, transforms these reactive carbonyl compounds into highly valuable building blocks for the construction of more complex structures. In that context, a wide variety of *N*, *S*, and *O*-heterocycles have been accessed using protocols involving α-haloketones [1,2,3,4,5,6,7,8,9,10,11]. These compounds have also proven to be important intermediates in the synthesis of various organometallic species. [12,13,14]. Other applications have demonstrated their utility as key synthetic intermediates for pharmaceutical blockbuster compounds (Figure 1) [15,16,17,18,19,20,21,22,23,24,25,26,27].

In recent years, a significant body of work has been dedicated to the design and development of new synthetic protocols to access α-haloketones. Even though these processes have been reviewed in the literature, a thorough and comprehensive survey is still lacking. For example, recent reviews have dealt with the α-bromination [28,29] and α-iodination [30] of carbonyl compounds; however, these were very specific and only involved pathways towards a very limited range of α-haloketones. In 2018, Verkariya and co-workers reviewed the synthetic access to α-iodocarbonyl compounds [31]. Although a broad range of iodinated products were considered, the work was restricted to direct iodination routes. In 2003, Erian and co-workers reported a survey involving the synthetic access to and application of α-haloketones [32]. Here, again, synthetic protocols yielding the desired ketones were only briefly discussed and the authors relied on the work published by De Kimpe and Verhé [33]. An up-to-date review of the synthetic access to α-haloketones is clearly needed. Therefore, the present contribution aims to describe and evaluate the diverse protocols leading to α-haloketones that have been published over the past two decades. Since the number of reports in the literature is quite significant, this review will be limited to methods generating carbonyl compounds carrying one halogen atom at their α-position, and will mainly focus on aromatic α-haloketones compounds, as these appear to have the highest synthetic value [34,35,36].

## 2. Direct α-Halogenation of Aryl Ketones

**Scheme 1:**
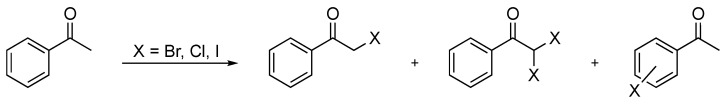
Moderate selectivity depicted by some direct halogenation protocols.

α-Haloketones are most commonly obtained from their corresponding carbonyl analogues through direct halogenation. Over the years, a variety of direct halogenation routes have been developed with apparent advantages and disadvantages. The readily available starting material for these reactions and the overall reaction efficacy have rendered these processes highly useful for synthetic chemists. At the same time, these direct halogenation reactions exhibited certain drawbacks, such as moderate conversions, long reaction times, cumbersome procedures and the use of hazardous and toxic reagents and solvents. In addition to the desired α-haloketone, some protocols also yielded the α,α-dihalogenated and/or aromatic ring halogenated by-products (Scheme 1). This moderate selectivity consequently complicates the purification process.

### 2.1. Electrophilic Halogenation

#### 2.1.1. Br_2_ as Halogenation Agent

**Scheme 2:**
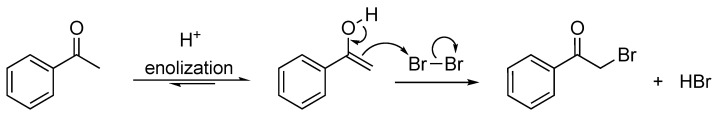
Acid-promoted bromination mechanism.

The most straightforward route to α-haloketones involves the reaction of enolizable aromatic ketones with electrophilic X_2_, under acidic (or basic) conditions; these conditions are typically needed to generate the nucleophilic enol (Scheme 2).

**Figure 2:**
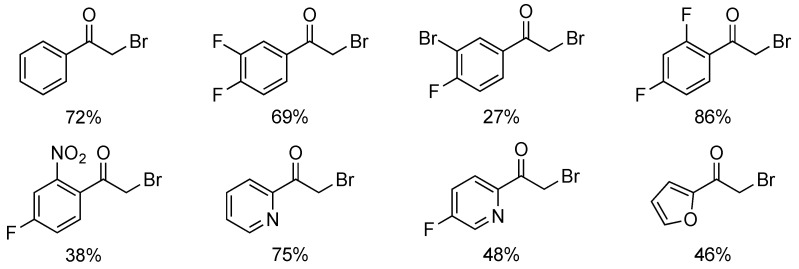
Scope of MW radiated bromination reported by Li and Xiang.

The procedure developed by Li, Xiang and co-workers in 2014 enables the bromination of aryl ketones under acidic conditions [37]. A small excess of Br_2_ (1.1 equiv.) was added to a solution of ketone in glacial HOAc and subsequently irradiated for 5 h in a microwave (MW). Figure 2 presents the bromination scope with respective isolated yields. The main drawback of this procedure is the use of HOAc as a solvent, which substantially limited the substrate scope; acid-sensitive functional groups (e.g., -OH, -NH_x_) cannot tolerate these reaction conditions.

**Scheme 3:**
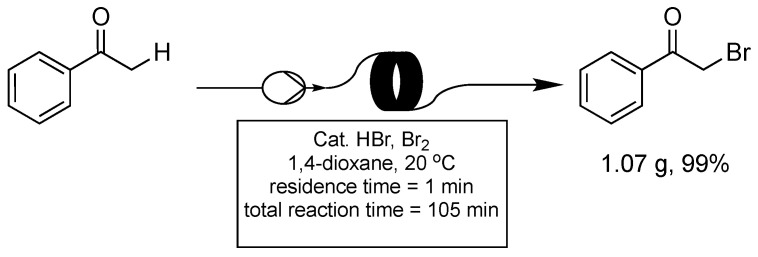
Continuous flow procedure for the α-bromination of acetophenone.

Using a similar strategy, Nieuwland developed a continuous flow procedure for the α-bromination of acetophenone [38]. 2-Bromo-1-phenylethanone was isolated in 99% yield upon treatment of the substrate with HBr and bromine in 1,4-dioxane (Scheme 3). The authors claimed that ring brominated or dibrominated products could not be observed, which highlights the excellent selectivity of the process. This work showcased a highly effective α-bromination protocol which could be applicable on an industrial scale. However, the large-scale use of toxic and corrosive HBr and Br_2_ reagents would require important safety measures to avoid leakage or contact of the reagents with the environment/operators, which remains a significant drawback.

**Scheme 4:**
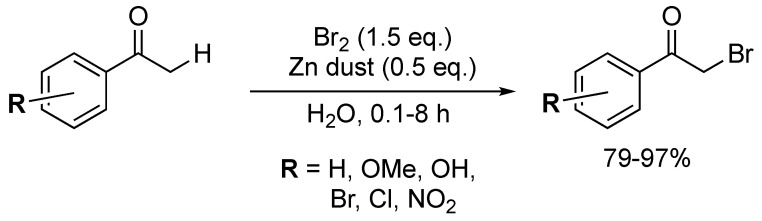
ZnBr_2_-mediated α-bromination of aromatic carbonyls.

Attempts to substitute the Bronsted acid activation with a Lewis acid activation have shown promising results, via the in situ formation of ZnBr_2_ from Zn dust and bromine (Scheme 4) [39]. The aqueous procedure was well tolerated by substituents (i.e., *ortho*) close to the carbonyl centre. However, the use of toxic metallic Zn and Br_2_ counteracted the progress made in terms of a greener approach [40,41].

**Scheme 5:**
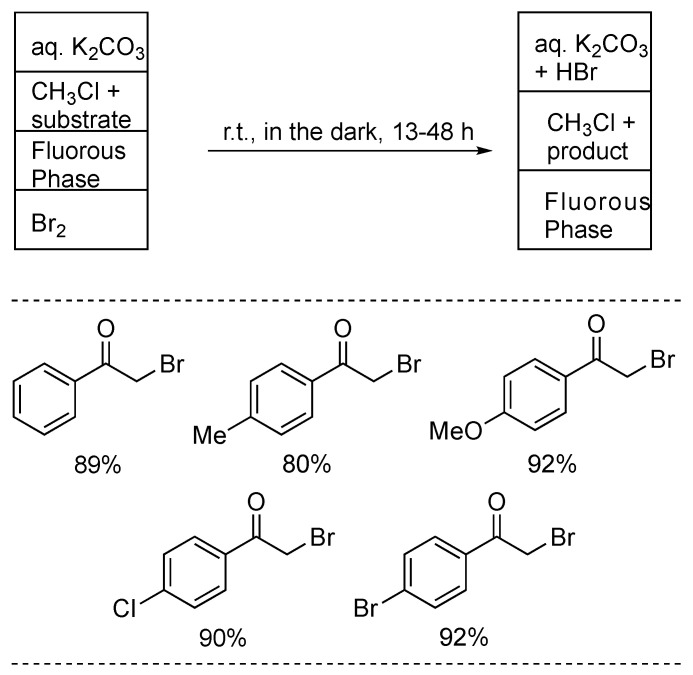
Phase-vanishing α-bromination.

Another attempt to alleviate the acid sensitivity of substrates/products was developed by Ryu and co-workers, using a phase-vanishing protocol (Scheme 5) [42]. The reaction mixture consisted of four separate phases: (i) an aqueous phase, to trap HBr, (ii) an organic (CH_3_Cl) phase (Galden HT135), containing the substrate, (iii) a fluorous phase, and (iv) the bromine phase. The fluorous phase acted as a membrane to separate the Br_2_ and the substrate-containing phase. Upon stirring, the fluorous phase enables the former to mix with the latter phase. During the reaction, HBr migrates to the aqueous layer, and at the end of the reaction, the bromine phase vanishes. This procedure thus prevented possible product decomposition induced by HBr, as the substrate and the acid were contained in different phases. This quadruple phase method simplified the purification of the desired α-bromoketones. In total, five α-bromoketones were isolated in good to excellent yields in this manner. It should be noted that dibrominated products were observed in trace amounts, which could complicate purification.

**Scheme 6:**
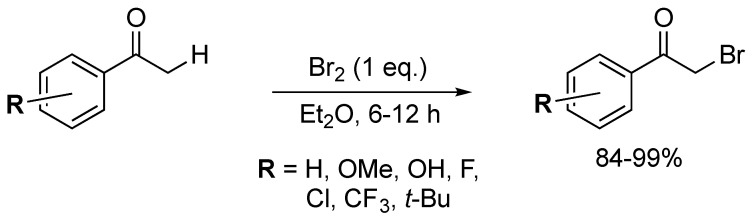
Acid-free α-bromination of aromatic carbonyls by Chen et al.

Chen et al. improved the reaction by successfully performing the α-bromination of a very broad substrate scope (21) in good to excellent yields (84–99%) without any additional activator (Scheme 6) [43]. The presence of both electron-withdrawing (e.g., Cl, Br, CF_3_) and -donating (e.g., OMe, *t*-Bu, OH) groups was well tolerated. Moreover, this route’s efficacy was not significantly affected by the presence of steric bulk at the *ortho*-position.

In 2014, Krupadanam and co-workers published an acid-free, Br_2_-mediated halogenation [44]. One equivalent of bromine was added dropwise to an ice-cold solution of the substrate in diethyl ether, after which the solution was stirred for 2 h at room temperature. Eight 2-bromoacetophenones were isolated in moderate to good yields (Table 1). In addition, the procedure generated hetero-aromatic bromoketones, i.e., 2-bromo-1-(pyridin-3-yl)ethenone and 2-bromo-1-(thiophen-2-yl)ethanone, in moderate yields (70 and 76%, respectively). However, for the former, the bromination only proceeded upon addition of an acidic promotor (HBr or HOAc).

**Scheme 7:**
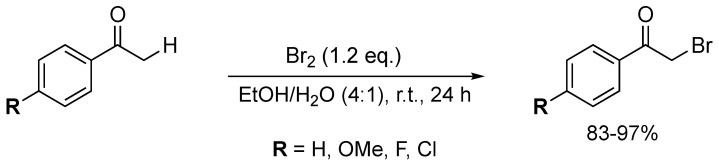
Acid-free α-bromination of aromatic carbonyls by Portilla and co-workers.

In 2018, Portilla described the bromination of four aromatic ketones mediated by bromine [45]. The corresponding α-bromoketones were isolated in good to excellent yields (Scheme 7). Krupadanam’s and Portilla’s routes stand out from other Br_2_-mediated halogenation reactions, as they demonstrated that the reaction can proceed without any promotor or catalyst. Additionally, the acid-free routes allowed a significant improvement in the halogenation yield.

All the above Br_2_-mediated methods are characterised by the same intrinsic drawbacks. These routes depict a moderate atom economy, since only one of the two available Br-atoms is incorporated into the final product. The “excess” bromine atom will interact with the α-H atom of the substrate, leading to corrosive and toxic HBr formation, inducing possible product decomposition. Furthermore, the toxic, irritating, and corrosive nature of bromine limits the applicability of a Br_2_-mediated route [46,47].

#### 2.1.2. I_2_ as Halogenation Agent

**Scheme 8:**
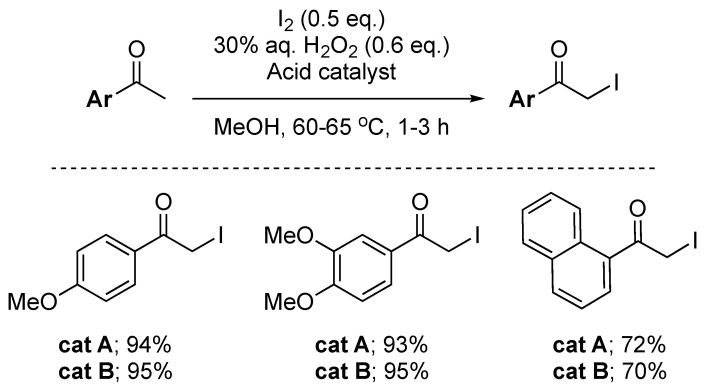
Scope of the I_2_, H_2_O_2_- and H_2_SO_4_/POM-mediated iodination.

In the direct iodination of ketones, Stavber and co-workers established an effective iodination route consisting of I_2_ (0.5 equiv.) and 30% aqueous H_2_O_2_ (0.6 equiv.) in methanol [48]. This system was only effective in the presence of an acid catalyst. The authors compared the efficacy of two catalytic systems; H_2_SO_4_ (**cat A**; 0.1 equiv.) and H_4_SiO_4_·12WO_3_·26H_2_O (POM) (**cat B**; 0.06 equiv.). Using both methods, three aromatic α-iodoketones were obtained in good to excellent yields (Scheme 8). The excellent iodine atom economy of this route should be stressed, as every I-atom is incorporated in the substrate. This method prevented the long-term persistence of harmful HI in the reaction mixture.

**Scheme 9:**
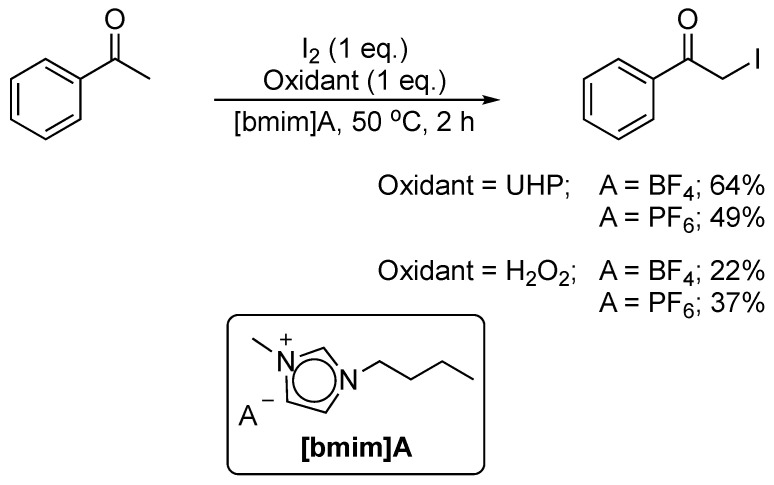
I_2_/H_2_O_2_- and I_2_/UHP-mediated iodination in Ils.

In 2007, Stavber updated the procedure by substituting the aqueous H_2_O_2_ with the solid urea–H_2_O_2_ (UHP) complex. This yielded a solvent-free direct iodination, though an increase in the I_2_ content from 0.5 to 1 equivalent was required [49]. The excellent iodine atom economy was thus not maintained. Furthermore, the iodination yields (22–58%) dropped significantly compared to those obtained from the previous route. Subsequently, it was demonstrated that both the I_2_/H_2_O_2_- and I_2_/UHP-mediated iodination could be performed in an ionic liquid (IL), thus easing the purification process [50]. The reaction was performed in both water-miscible and water-immiscible Ils, respectively, 1-butyl-3-methyl imidazolium tetrafluoroborate ([bmim]BF_4_) and 1-butyl-3-methyl imidazolium hexafluoro-phosphate ([bmim]PF_6_) (Scheme 9). Experimental data demonstrated that the substrates were less effectively iodinated in both Ils, compared to the solvent-free [49] and MeOH iodination protocols [48].

**Scheme 10:**
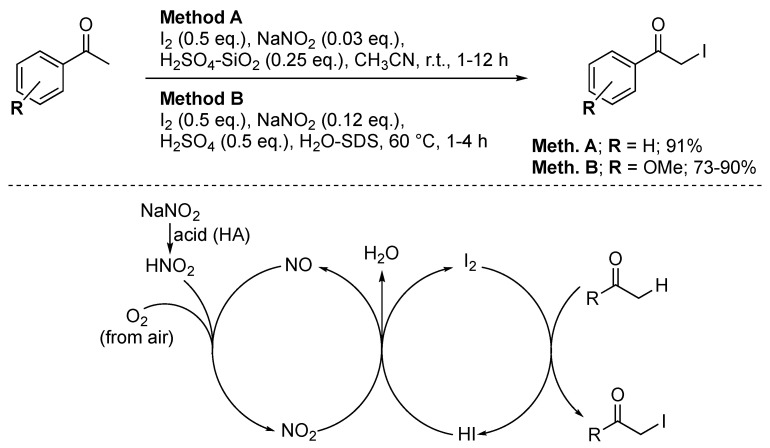
Stavber’s NaNO_2_-catalysed α-iodination reaction and mechanism.

Additionally, Stavber reported a sodium nitrite-catalysed procedure (Scheme 10, method A) [51]. NaNO_2_ is activated via treatment with silica-supported sulfuric acid. After activation, HNO_2_ is obtained, which is then oxidised by air to NO_2_. Since only 0.5 equiv. of I_2_ is used, after depletion of all I_2_ molecules, the obtained HI will then react with NO_2_ to reform I_2_ and NO; the latter is then again oxidised to NO_2_ by air. In this manner, 100% of iodine atom economy was achieved as only 0.5 equivalent of iodine was required for the reaction to proceed to completion. The sustainability of this route was further demonstrated, as air was used as the stoichiometric oxidant. Compared to other iodination routes, the purification was more straightforward as it only required separation from the inorganic salts. Due to the inherent health hazards linked to SiO_2_ [52,53,54], this procedure was later modified (Scheme 10, method B) [55]. The iodination was carried out in an aqueous sodium dodecyl sulphate (SDS) solution and promoted by H_2_SO_4_. The SDS micelles acted as an ideal promotor of the α-iodination of aryl ketones. This route displayed excellent selectivity as side chain functionalisation was exclusively obtained. This could be shifted towards aromatic ring iodination by changing the reaction medium to acetonitrile. The 100% iodine atom economy and the use of an aqueous reaction medium showcased the promising potential of the SDS-promoted route. From an economic point of view, this route is particularly interesting as it employed cheap and abundant reagents. Nonetheless, the presence of H_2_SO_4_ in the reaction medium still limited the substrate scope, as acid-sensitive functional groups (e.g., OH) are not compatible with these experimental conditions.

**Scheme 11:**
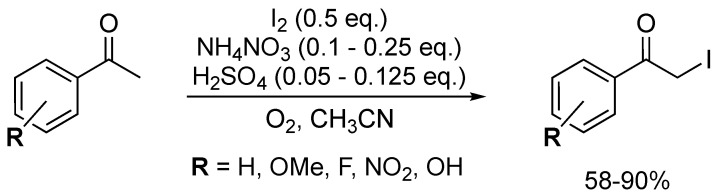
NH_4_NO_3_ and I_2_-based direct halogenation protocol.

In 2014, an alternative method was developed using NH_4_NO_3_ (0.1–0.25 equiv.), I_2_ (0.5 equiv.), H_2_SO_4_ (0.05–0.125 equiv.) and air in CH_3_CN (Scheme 11) [56]. Under these conditions, several ketones were iodinated in moderate to excellent yields (58–90%). In addition, OH-substituted aromatic ketones were successfully transformed into their iodinated analogues. However, for di- and tri-methoxy-substituted ketones, selective ring iodination instead of α-iodination occurred; these by-products account for a required and more tedious purification process.

**Scheme 12:**
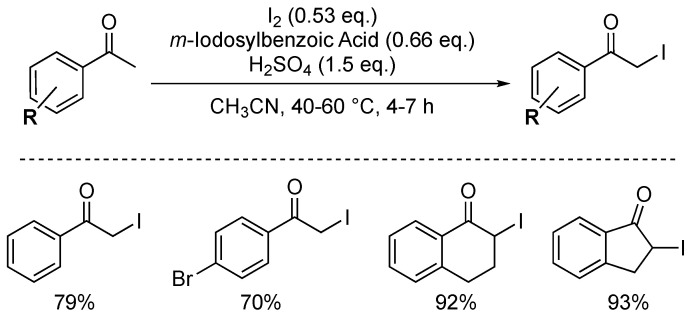
*m*-Iodosylbenzoic acid- and I_2_-mediated iodination.

Alternatively to O_2_ and H_2_O_2_, Zhdankin and co-workers demonstrated that the hypervalent *m*-iodosylbenzoic acid could also act as a suitable oxidation agent [57]. With the reaction conditions shown in Scheme 12, a variety of aromatic and aliphatic ketones and β-dicarbonyls were iodinated. At the end of the reaction, the reduced form of the hypervalent iodine oxidant, *m*-iodobenzoic acid, could be easily removed by passing the reaction mixture through an anionic exchange resin (Amberlite IRA 900 HCO_3_^−^). Afterwards, recovery of the *m*-iodobenzoic acid was achieved by treating the resin with HCl. With similar drawbacks to earlier strategies, this method still allowed for 100% I-atom economy while also carrying the benefits of recycling the oxidizing reagent. 

**Scheme 13:**
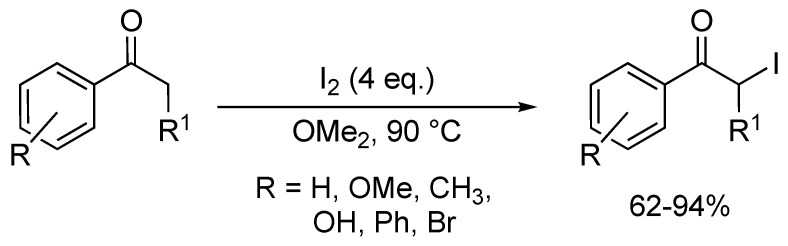
Iodination system developed by Rao and co-workers.

Over the past two decades, numerous acid-free direct iodination protocols have been developed. These methods tolerated acid-sensitive functional groups, such as amines or alcohols. A remarkable iodination system was developed by Rao and co-workers, which only required the addition of iodine (4 equiv.) to the methyl ketone in dimethylether at 90 °C (Scheme 13) [58]. Several aromatic α-iodoketones were obtained in moderate yields (62–94%). In this reaction, the dual role of I_2_ was highlighted as it acts as both a Lewis acid and a direct iodine source. The acid-free conditions and short reaction times were this route’s main advantages. However, the use of a highly volatile solvent, the excess of I_2_ and the inevitable generation of HI negatively impacted this direct iodination route. The corrosive nature of HI involves extra hazards and might lead to product decomposition.

**Scheme 14:**
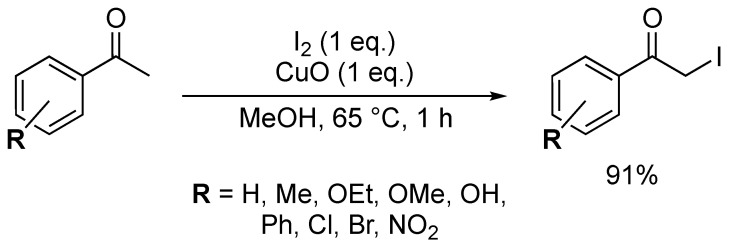
Copper-mediated iodination of ketones by Yin and co-workers.

Using neutral conditions, Yin and co-workers reported a metal-mediated iodination of ketones through the addition of CuO (1 equiv.) and I_2_ (1 equiv.) to the substrate in MeOH (Scheme 14) [59]. The presence of electron-donating (e.g., OMe, Me) and electron-withdrawing substituents (e.g., Cl, Br) on the aryl ketones was well tolerated. The corresponding α-iodoketones were obtained in 83–99% yield. For nitro-substituted aryl ketones, the yield decreased significantly to 53% and formation of the dimethyl ketal product occurred. This protocol stands out due to the use of inexpensive reagents and its excellent iodination yields. Nonetheless, a significant amount of harmful HI was generated.

**Scheme 15:**
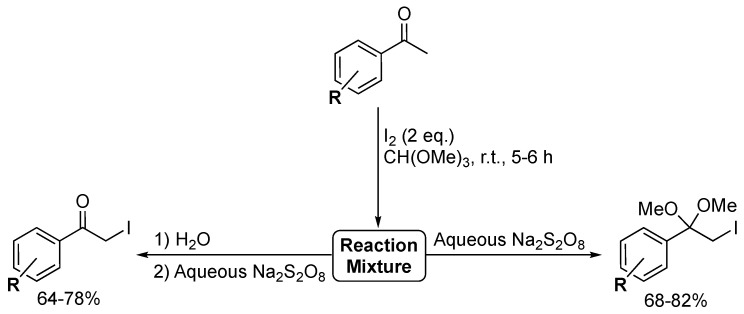
Iodine- and trimethylorthoformate-mediated iodination.

An iodine- and trimethylorthoformate-mediated iodination was reported in 2008 (Scheme 15), yielding several aromatic ketones with a wide range of substituents (i.e., *para*-OH, -Me, -F and common protecting groups such as -OBn, -OTBS and -OAc) in good yields (64–78%) [60]. The authors claimed that the work-up drastically altered the outcome of the reaction, since the true reaction mixture contained primarily the α-iodinated dimethoxy ketal product. Upon reaction completion, when the reaction was directly treated with an aqueous Na_2_S_2_O_3_ solution followed by extraction, the α-iodoketal product was mainly obtained (Scheme 15, right). However, if the reaction mixture was first stirred in water, then treated with the Na_2_S_2_O_3_ solution, α-iodoketones were obtained as the main products (Scheme 15, left).

**Scheme 16:**
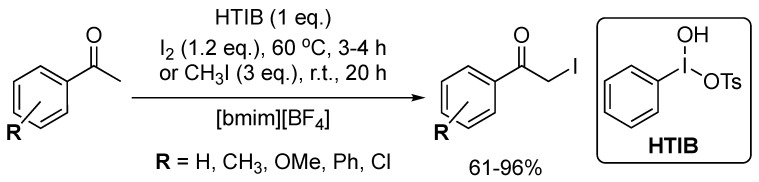
The halogenation of ketones by Lee and co-workers.

In 2010, Lee and co-workers illustrated the halogenation of ketones using another iodine activator, the hypervalent [hydroxyl(tosyloxy)iodo]benzene (HTIB), in an IL, i.e., [bmim][BF_4_] (Scheme 16) [61]. Two different iodination agents, iodine (1.2 equiv.) and methyl iodide (3 equiv.), were compared for a set of ketones. Although both generated the corresponding α-iodoketones in similar yields, they required different reaction conditions. The I_2-_mediated direct halogenation was carried out at 60 °C for 3–4 h. With methyl iodide, the reaction time increased to 20 h and the temperature decreased to room temperature. The protocol yielded five α-iodinated aryl ketones in moderate to excellent yields (61–96%). This route emerged as a relatively mild and green direct halogenation route linked to HTIB, a neutral, highly stable, and relatively safe oxidant. The use of an IL also contributed to simplifying the work-up process by allowing the product to be simply extracted without any further purification needed.

Goswami reported the selective α-iodination route mediated by iodine (0.5 equiv.) and Oxone^®^ (2KHSO_5_·KHSO_4_·K_2_SO_4_) (0.1 equiv.) [62]. The reaction proceeded by grinding the reagents and the ketone in a mortar with a pestle. A diverse set of 1,3-dicarbonyls and ketones were halogenated in excellent yields. However, only a limited number of aromatic ketones were tested under these conditions to afford the desired products in excellent yields (91%), after grinding for 3 min. The procedure stands out from other direct iodination routes due to its solvent-free approach, involving a more straightforward work-up. So far, the very limited scope of aryl ketones can be considered as its major weakness.

**Scheme 17:**
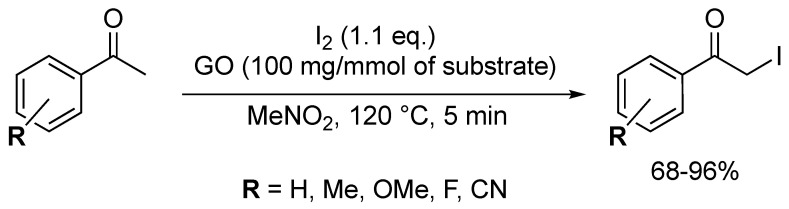
GO-catalysed iodination of ketones.

In 2018, Gong and co-workers designed a graphene oxide (GO)-catalysed iodination of arenes and ketones in nitromethane (Scheme 17) [63]. Upon the addition of I_2_ (1.1 equiv.), several aryl ketones were efficiently iodinated when stirred for 5 min (min) at 120 °C. Substrates bearing electron-donating groups (i.e., OMe and Me) performed well under these conditions (96% for both). In comparison, substrates with electron-withdrawing substituents (i.e., F and CN) gave somewhat lower yields (74% and 68%, respectively). The reaction proceeded via a radical pathway, with an iodine radical being formed under the influence of the unpaired electrons of the GO; single electron transfer (SET) from the iodinated substrate back to the GO regenerated the GO catalyst and allowed its re-entry into the catalytic cycle. The authors were able to scale up the reaction to the gram scale in air, thus adding a major benefit to this approach. Despite the efficiency and high applicability of this protocol, the generation of stoichiometric amounts of HI and the high temperature needed are considered major drawbacks.

Overall, direct halogenation methods, employing Br_2_ or I_2_, possess similar intrinsic weaknesses. Bromine is a toxic, corrosive and irritating halogenation agent [46,47]. Although less harmful than bromine, the use of iodine also poses serious safety and health issues [64,65]. Hence, the next sections will focus on halogen sources that are generally less harmful to human health and to the environment.

#### 2.1.3. NXS as Halogenation Agents

**Figure 3:**
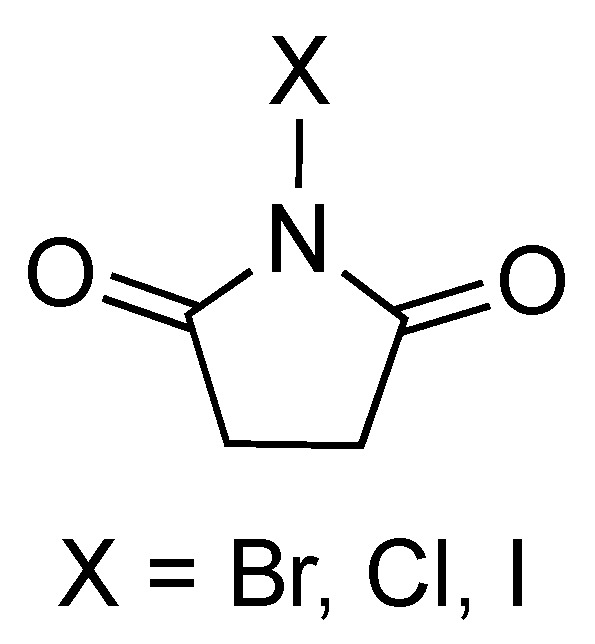
Structure of *N*-halosuccinimide.

The user friendliness, availability, and low cost of *N*-halosuccinimides (NXS) has enabled its widespread use as a halogenation reagent (Figure 3). NXS can be recycled after the reagent is consumed in the halogenation reaction, demonstrating its sustainable nature. 

**Figure 4:**
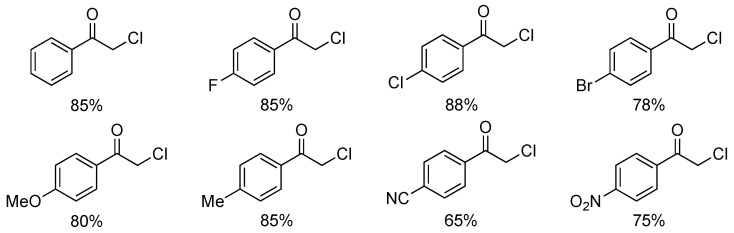
Chlorination scope by Lim and co-workers.

In 2017, Lim and co-workers reported a direct bromination and chlorination route optimised for aromatic ketones [66]. The procedure involved the addition of NBS (1.05 equiv.) and TMSOTf (0.05 equiv.) to the starting material and stirring the obtained solution in MeCN for three days at room temperature. Five α-bromoketones (i.e., *p*-H, -F, -Cl, -CN and -NO_2_) were isolated in moderate to good yields (60–77%). Despite the straightforward work-up procedure, the extremely long reaction times have limited the widespread use of this protocol. Chlorination was also established via the addition of NCS (1 equiv.) and *para*-toluenesulfonic acid-monohydrate (*p*TsOH·H_2_O) (1.5 equiv.) to the ketone. After stirring for 7 h at 80 °C, eight α-chloroketones were obtained (Figure 4). The excess of *p*TsOH·H_2_O used renders the protocol incompatible with acid-sensitive groups. 

**Scheme 18:**
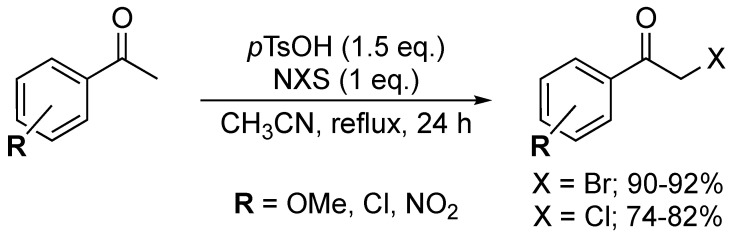
*p*TsOH-based chlorination and bromination by Lee and co-workers.

Similar *p*TsOH-based chlorination and bromination were also described by Lee and co-workers (Scheme 18) [67]. In these reactions, 1.5 equiv. of the acid and 1 equiv. of NXS yielded three α-bromo (90–92%) and three α-chloroketones (74–82%) with three different substituents on the aromatic ring (i.e., *p*-OMe, -Cl, -NO_2_). Here, again, the very limited scope puts the general nature of the method into serious doubt.

A more general *p*TsOH-catalysed bromination protocol was developed by Huang and co-workers using MW irradiation [68]. A solution of the ketone, NBS (1 equiv.) and *p*TsOH (0.1 equiv.) in dichloromethane (DCM) was irradiated for 30 min at 80 °C. This procedure yielded 11 aromatic α-bromoketones in good to excellent yields (85–95%). The presence of electron-withdrawing, -donating or acid-sensitive substituents (i.e., OH) on the aryl ketones was well tolerated. The position of these substituents on the aromatic ring did not influence the bromination efficiency. The versatility of this route was further highlighted by the bromination of heteroaromatic ketones with high efficacy (93–95%).

**Scheme 19:**
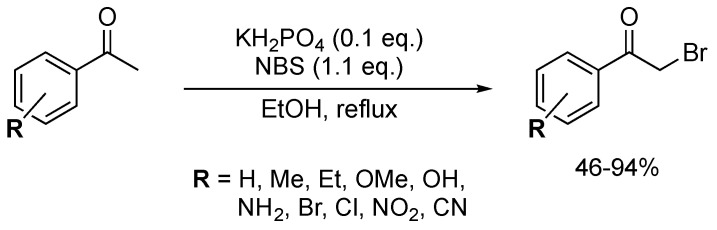
Direct bromination of aryl ketones as reported by Reddy and co-workers.

KH_2_PO_4_ could also promote the direct bromination of aryl ketones as reported by Reddy and co-workers (Scheme 19) [69]. Under mild acidic conditions using 0.1 equiv. of KH_2_PO_4_ and 1.1 equiv. of NBS, a wide variety of brominated products were isolated in moderate to excellent yields (46–94%). Nitro- and cyano-substituted aromatic ketones were less efficiently brominated. For substrates bearing OH and NH_2_ substituents, ring bromination was exclusively observed.

**Scheme 20:**
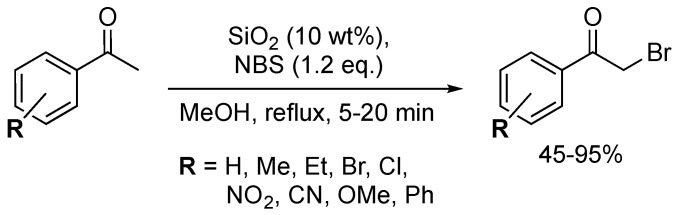
SiO_2_-catalysed bromination of aryl ketones.

Moving away from Brønsted acid-based conditions, the same group highlighted silica as another efficient promoter for the bromination of aryl ketones [70]. Using the conditions depicted in Scheme 20, a wide variety of aryl ketones was successfully brominated. Substrates bearing highly electron-donating (i.e., *p*-OMe) and electron-withdrawing (i.e., NO_2_, CN) groups did not fare well in this protocol, as their corresponding α-bromoketones were obtained in yields of 64%, 42% and 45%, respectively. For some substrates, dibromination also occurred, highlighting the procedure’s modest selectivity. The short reaction times, however, can be considered as the main feature of this route.

It should be mentioned that SiO_2_-catalysed α-bromination is not a new concept. Paul and co-workers had previously reported the NBS- and HClO_4_·SiO_2_-mediated halogenation affording 11 aromatic α-bromoketones in good to excellent yields (77–93%) [71]. Using this process, heteroaromatic (i.e., pyridinyl, thienyl, furyl) ketone substrates were efficiently α-brominated, albeit with longer reaction times (16–18 h) compared to their aromatic counterparts (8–12 h). Similarly to the aforementioned routes, the protocol demonstrated a rather moderate selectivity as the α,α-dibrominated products were observed in some cases. Other protocols involving the use of modified SiO_2_, such as NaHSO_4_·SiO_2_ and HSO_3_·SiO_2_, have also demonstrated good catalytic activity in the direct halogenation reaction. Both yielded 2-bromo-1-phenylethanone in 72% and 87% yield, respectively. The authors claimed that HSO_3_·SiO_2_ could even be recycled and reused for three consecutive runs without significant loss in catalytic activity. Additionally, Salama reported that SiCl_4_ efficiently catalysed the direct halogenation of aromatic ketones [72]. In this procedure, SiCl_4_ (2 equiv.) and NXS (2 equiv.) were added to the aryl ketone in CH_3_CN. The protocol yielded several α-chloro-, α-bromo- and α-iodoketones in moderate to excellent yields.

**Scheme 21:**
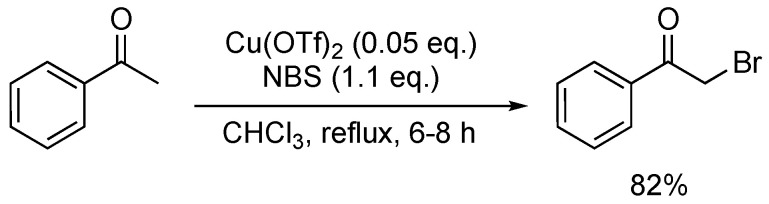
Cu(OTf)_2_- and NH_4_OAc-catalysed bromination.

Other Lewis-acid-based protocols involved the use of transition metals to efficiently perform the direct halogenation of aryl ketones [73]. Sudalai and co-workers described a Cu(OTf)_2_-catalysed bromination reaction that afforded α-bromoketones in 65–89% yield (Scheme 21). Next to the mono-brominated product, α,α-dibromoketones were also observed as a minor product, making this route less attractive compared to existing and more selective α-bromination procedures.

Waghmode and Ramaswamy designed a photochemical α-bromination of cyclic and acylic ketones. This route generated seven aromatic bromoketones upon the addition of NBS (1.05 equiv.) to the substrates and subsequent UV-VIS irradiation in diethylether [74]. Thorough assessment of this route’s efficiency is not possible, as bromination yields were not reported. The authors mentioned that α,α-bromination occurred for most of the substrates, which highlights the modest selectivity of the developed procedure.

**Scheme 22:**
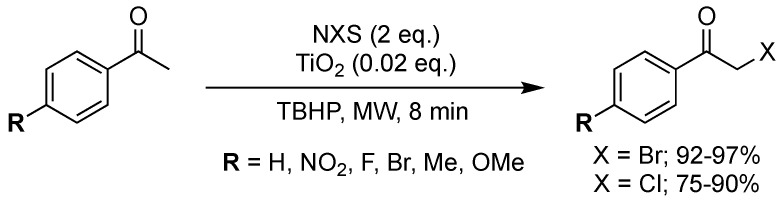
Jangdid’s NXS-mediated direct bromination and chlorination.

In 2019, Jangid and co-workers developed a TiO_2_ nanoparticle (NP)-catalysed bromination and chlorination of aromatic ketones [75]. With the corresponding NXS and *t*-butyl hydrogen peroxide (TBHP), 12 α-haloketones were obtained in good to excellent yields (Scheme 22). The short reaction times and excellent halogenation yields can be considered as the main advantages of this route.

**Figure 5:**
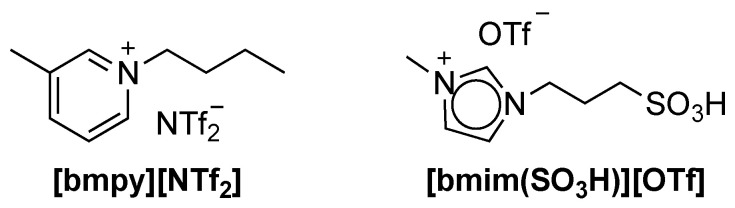
Ionic liquids as halogenation solvents.

The use of volatile and hazardous organic solvents counteracted the progress that some NXS-based methods accomplished in terms of sustainability. Thus, Togo and co-workers attempted to provide a greener bromination approach [76]. A set of ketones was halogenated upon addition of NBS (1.2 equiv.) and *p*TsOH·H_2_O (0.2 equiv.) in two different ILs, i.e., [bmim][PF_6_] and *N*-butyl-*N*-methyl-pyrrolidinium bis(trifluoromethanesulfonyl)imidate ([bmpy][NTf_2_]) (Figure 5, left). The use of an IL not only facilitated the work-up, but also allowed the reaction medium to be reused up to seven times without significant loss in bromination efficiency. Both ILs appeared to be effective halogenation reaction media, as eight aromatic α-haloketones were obtained in good to excellent yields (74–96%). 

**Scheme 23:**
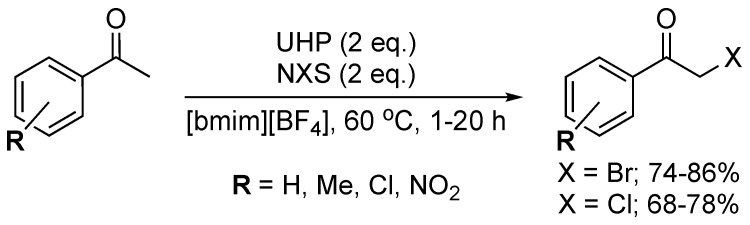
UHP/NXS α-bromination in [bmim]BF_4_.

Lee and Park designed an alternative procedure for the α-bromination in an IL, i.e., [bmim]BF_4_ (Scheme 23) [77]. The UHP (2 equiv.)- and NXS (2 equiv.)-mediated reaction yielded four α-chloro and four α-bromoketones (*p*-H, -Me, -Cl and -NO_2_) in moderate to good yields (68–78% and 74–86%, respectively).

Another NXS-mediated halogenation in an IL, i.e., 1-methyl-3-(4-sulfobutyl)imidazolium triflate ([bmim(SO_3_H)][OTf]), was also developed by Stavber (Figure 5, right) [78]. Remarkably, the selectivity of the procedure was highly dependent on the type of halogenation carried out. In general, α-iodination of aromatic ketones was exclusively obtained, while a mixture of α- and α,α-dihalogenated product was observed for the bromination, with the former species as the major product. Upon increasing the reaction time, this ratio could be shifted to selectively obtain the latter product. The chlorination exclusively yielded the dihalogenated aryl ketones. The authors claim that [bmim(SO_3_H)][OTf] could be recycled and reused for up to eight consecutive runs without any observable loss in halogenation efficiency. The sustainable character of this route and its versatility make it stand out from other halogenation routes.

**Scheme 24:**
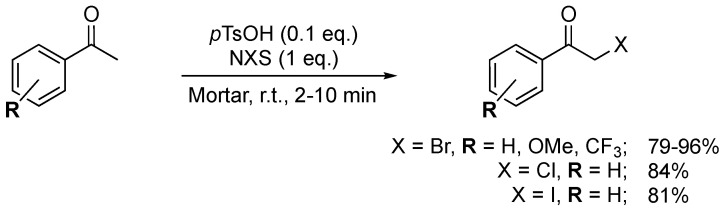
Solvent-free halogenation of aromatic ketones.

Achieving a good reaction performance under solvent-free conditions is an important step forward for any reaction and is good practice for future advancement in sustainable chemistry. Stavber reported the solvent-free halogenation of acetophenone derivatives, mediated by sub-stoichiometric amounts of *p*TsOH (Scheme 24) [79,80]. The reagents were ground in a mortar with a pestle, yielding α-bromo, α-chloro and α-iodoketones in satisfactory yields. This procedure could be applied to the bromination, chlorination, and iodination of ketones. In the case of the chlorination, dichlorination of the substrates was a serious issue. Note that thus far, this dichlorination issue has been mentioned several times. Furthermore, trimethoxy-substituted ketones generated exclusive ring bromination. Interestingly, performing the bromination reactions in an aqueous solvent shifted the selectivity of this route toward ring bromination.

In 2003, Lee and co-workers published a solvent-free iodination of aromatic ketones mediated by NIS (1.2 equiv.) and *p*TsOH (1.2 equiv.) [81]. Five α-iodoketones were obtained within 1–1.2 min in good to excellent yields (75–90%) after irradiation of the reaction mixture in a microwave at 700 W. The system stood out from other direct iodination routes mainly due to its short reaction times and excellent performance.

In 2015, Patel and co-workers reported catalyst-free bromination in neat conditions [82]. Under these conditions, the ketone and NBS (1.1 equiv.) were ground in a mortar in order to obtain a homogenous solid mixture, which was subsequently irradiated in the MW at 300 W for 1–4 min. Ten α-bromoketones were obtained in good to excellent yields (82–93%). This protocol tolerated the presence of electron-donating and -withdrawing substituents on the aromatic ring. However, a significant decrease in bromination yield was observed for dimethoxy- and nitro-substituted aryl ketones. The recycling strategy of NBS, developed by Patel, the solvent- and catalyst-free nature as well as the short reaction times, highlighted the sustainable character of this methodology.

#### 2.1.4. Halides (X^−^) as X_2_ Sources

**Scheme 25:**
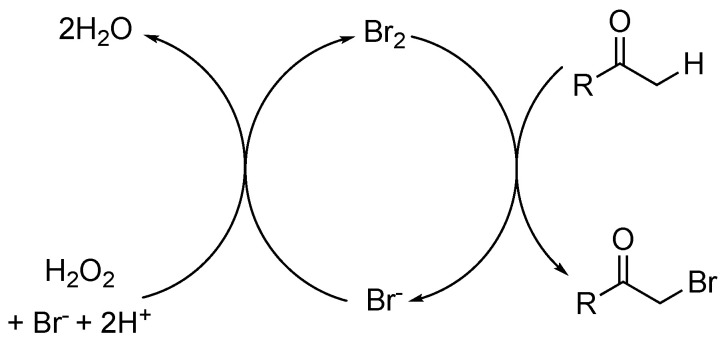
Mechanism of the direct electrophilic bromination of ketones using a Br^−^/H_2_O_2_ system.

Methods using halide anions (X^−^) are among the most promising methodologies as they offer a cheaper and safer alternative for direct electrophilic halogenation of ketones, especially when combined with environmentally benign oxidants, such as H_2_O_2_, molecular oxygen or even an electrochemical cell setup. Typically, these methods involve the use of MX, HX or NR_3_X in combination with an oxidant to generate in situ the active halogenation reagent, X_2_. An example using H_2_O_2_ in acidic media is shown in Scheme 25.

##### Metal Halides (MX)

**Scheme 26:**
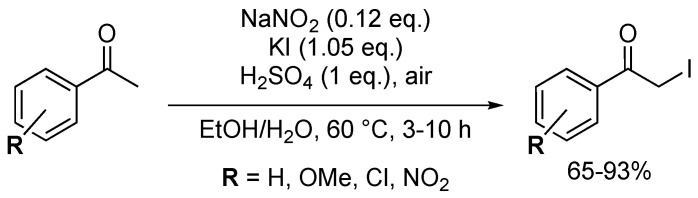
NaNO_2_-catalysed iodination of aromatic ketones.

Similarly to their NaNO_2_-I_2_ system (Scheme 25), Stavber and co-workers designed a NaNO_2_-KI-based procedure for the α-iodination of aromatic ketones, which yielded nine α-iodoketones in moderate to excellent yields (Scheme 26) [83]. KI exhibited similar iodination efficiency to molecular iodine when compared to the NaNO_2_-I_2_-mediated reaction [55]. Nonetheless, potassium iodide remains a much better choice than I_2_ as an iodination agent, as it is cheaper and safer to handle. It should be noted that a similar effect of the bromination solvent on the regioselectivity was observed, as described for the I_2_ reaction. When carried out in aqueous ethanol, selective α-bromination occurred. When the iodination was performed in dry acetonitrile, the aromatic ring was exclusively halogenated.

**Scheme 27:**
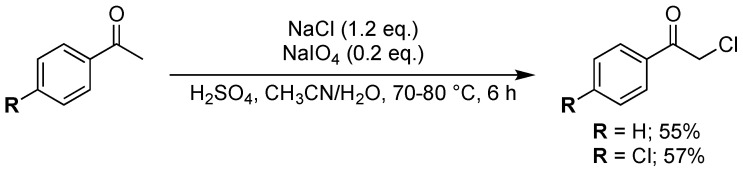
NaIO_4_-NaCl α-chlorination of aryl ketones.

Another MX-based procedure was developed by Zhang and co-workers using NaCl and K_2_SO_8_ to perform the chlorination of arenes in acetonitrile [84]. The selectivity of the method was altered to exclusively lead to the α-chlorination when phenylethanone was used. Since the scope only consisted of one aryl ketone, broader screening is required for a better assessment of this route’s efficacy. A similar sodium chloride-based halogenation was reported in 2004 (Scheme 27) [85], with a broad range of alkanes, arenes and ketones being converted into their chlorinated derivatives. However, the method only showed moderate chlorination efficiency with aryl ketones. 

**Scheme 28:**
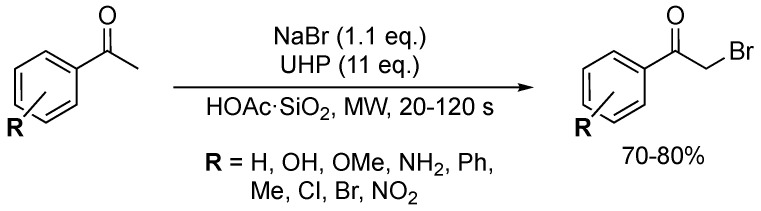
Gupta’s solvent-free NaBr-mediated halogenation.

In 2005, Gupta and co-workers reported the synthesis of α-bromoketones mediated by the combination of UHP and NaBr with acetic acid-functionalised silica. After microwave irradiation at 300 W, 12 α-bromoaryl ketones could be isolated in good yields (Scheme 28) [86,87]. The presence of electron-withdrawing, -donating and acid-sensitive groups on the phenyl ring was well tolerated. This route’s main features are its solvent-free conditions and the extremely short reaction times. 

##### Quaternary Ammonium Halides (NH_4_X)

**Scheme 29:**
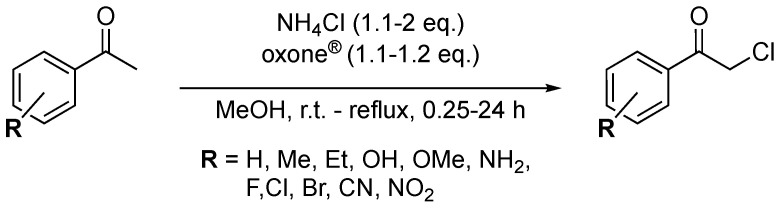
NH_4_Cl- and Oxone^®^-mediated halogenation.

Over the past two decades, a significant amount of work has been dedicated to the development of NH_4_X- and Oxone^®^-mediated halogenation reactions. The high stability, water solubility and non-toxic nature make Oxone^®^ an attractive oxidation agent. In 2012, Narender and Zhou independently described the chlorination of aromatic ketones in MeOH using a NH_4_Cl and Oxone^®^ system (Scheme 29) [88]. The mechanism follows a similar path to MX-based oxidation approaches, generating in situ the X_2_ active reagents. Both protocols established a broad substrate scope although Narender’s method showed the best chlorination efficiency. The two procedures reported a similar trend, i.e., electron-poor aryl ketones were less efficiently chlorinated. No polychlorination or ring chlorination was observed for either route, stressing their excellent selectivity. 

**Scheme 30:**
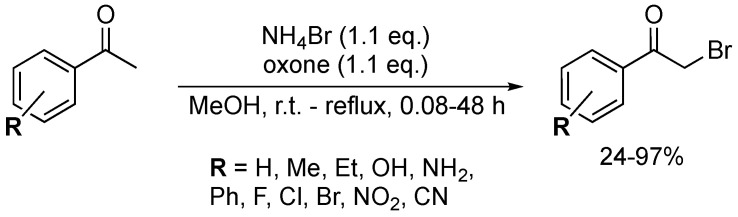
The Oxone-based bromination of aromatic ketones by Narender and co-workers.

Using the same system, Narender and co-workers reported the α-bromination of 21 aromatic ketones in low to excellent yields (24–97%) (Scheme 30) [89]. Electron-deficient substrates (i.e., bearing CN and NO_2_ groups) appeared to be deactivated under these conditions, as they were isolated in only 21 and 35% yield. Furthermore, this route showed a rather moderate selectivity for these substrates, since α-bromo-dimethylketals were obtained as the major products. Additionally, some substrates showed signs of ring halogenation, adding to the system’s selectivity issues.

**Scheme 31:**
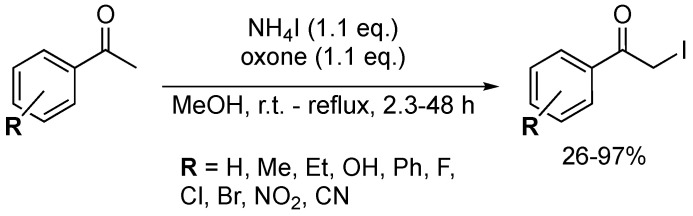
Oxone-based iodination of aromatic ketones by Narender and co-workers.

Narender and co-workers went on to develop another procedure for the α-iodination of ketones, generating a wide variety of α-iodoketones (Scheme 31) [90,91]. Again, a similar trend was observed; aryl ketones bearing electron-withdrawing groups were less efficiently iodinated. This route yielded a significant amount of either the ring iodinated or the α,α-diiodinated product for substrates carrying OH- and OMe-substituents. The iodination thus appeared to be less selective than the chlorination, which ultimately hinders its applicability.

**Scheme 32:**
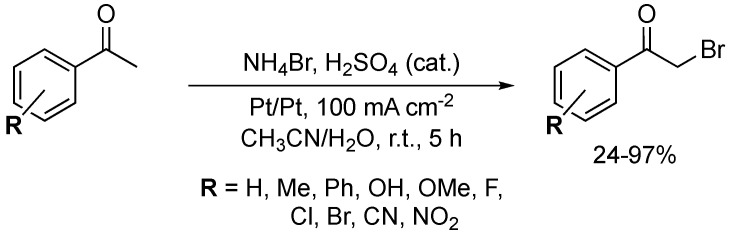
Electrochemical α-bromination of ketones.

One of the most interesting approaches employing ammonium halide salts is its combination with electrochemical oxidation conditions [92]. In an electrochemical cell equipped with a Pt/Pt electrodes, aryl ketones were efficiently brominated (Scheme 32). Under these conditions, Br^−^ was oxidised to Br_2_, which then reacted with water to generate the HOBr active species. The broad substrate scope highlighted the versatile nature of this protocol, and despite the acidic conditions, acid-sensitive groups (i.e., OH) were well tolerated. 

Makrandi and co-workers developed a solvent-free NH_4_Br-mediated α-bromination of alkanones [93]. After grinding a mixture of the starting material, NH_4_Br (2 equiv.) and (NH_4_)_2_S_2_O_8_ (2.5 equiv.) for 15 min at room temperature, 10 (hetero)aromatic ketones were isolated in good to excellent yields (83–92%). The procedure tolerated the presence of electron-withdrawing (i.e., Cl, NO_2_), -donating (i.e., OMe, Me) and acid-sensitive (i.e., OH) substituents on the aryl ketones. It should be noted that trace amounts of water were required to ensure complete homogenisation of the solid reaction mixture. 

##### Hydrogen Halides (HX)

At first sight, HX might seem a valuable halogenation alternative to bromine, as it will ensure a high halide atom economy. However, the highly corrosive and toxic nature of this reagent still limits its applicability [94]. Nonetheless, a significant body of work has been reported on this topic and several noteworthy advances have been made.

**Scheme 33:**
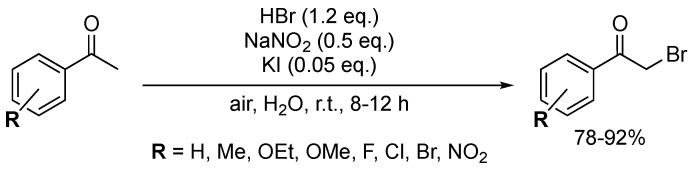
Aqueous HBr-mediated bromination by Akamanchi and co-workers.

Akamanchi and co-workers developed an aqueous HBr-based halogenation system for aromatic ketones [95]. In this system, the combination of NaNO_2_ and KI acted as the bromination catalyst (Scheme 33). The versatility of this route was demonstrated through the bromination of a broad range of aromatic ketones, bearing electron-withdrawing or -donating substituents.

**Scheme 34:**
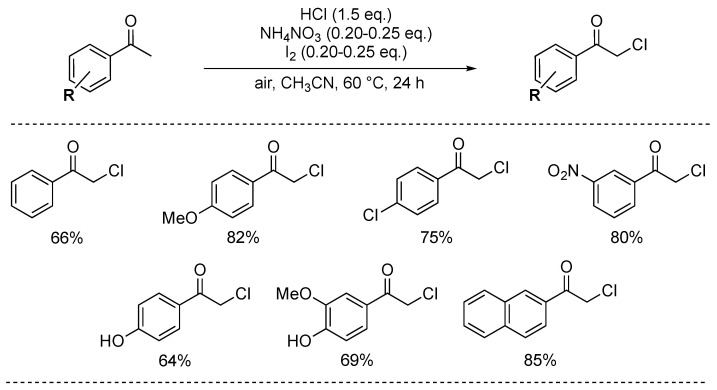
Stavber’s NH_4_NO_3_- and I_2_-catalysed chlorination.

Stavber and co-workers demonstrated the versatility of an NH_4_NO_3_- and I_2_-based system in air [96]. The procedure yielded seven α-chloroketones in moderate to good yields (Scheme 34). This protocol stands out due to its excellent selectivity, as no ring chlorination or dichlorination was observed.

**Scheme 35:**
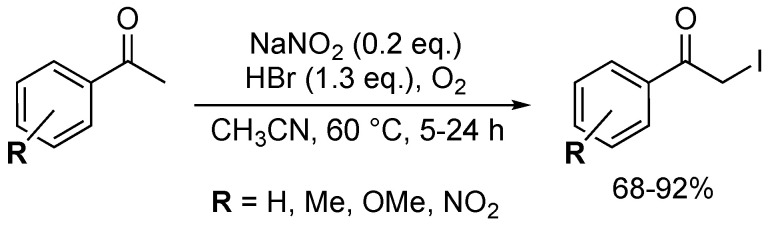
NaNO_2_- and O_2_-mediated bromination by Zhang and co-workers.

Zhang and co-workers developed an HBr, NaNO_2_ and O_2_-mediated ring bromination, yielding a diverse set of halogenated arenes (Scheme 35) [97]. When using aryl ketones, α-bromination occurred instead, generating five α-bromoketones in moderate to excellent yields (68–92%). The NO_2_-substituted aryl ketones underwent a less efficient bromination, which aligns well with previous observations on the deactivation of electron-poor substrates. The use of molecular oxygen as co-oxidant should also be highlighted.

**Scheme 36:**
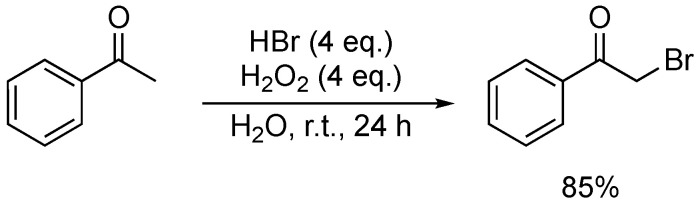
H_2_O_2_/HBr system by Stavber and co-workers.

The use of a more environmentally friendly oxidant system, such as H_2_O_2_, has also been reported in combination with HBr, by Stavber and co-workers (Scheme 36) [98]. Interestingly, the bromination was carried out in water, further highlighting the sustainability of this system. 1,3-Diketones, β-ketoesters, and cyclic ketones were brominated in good to excellent yields. However, the scope only consisted of one aromatic ketone, again not making it possible to perform an evaluation of the strength of the protocol. 2-Bromo-1-phenylethanone was isolated in 78% yield. This method exhibited moderate selectivity, since for some of the substrates, unwanted dibromination was a serious issue.

Wakharkar and co-workers performed a comparative study between a H_2_O_2_ and a TBHP system with HBr as the bromine source [99]. A set of substrates, bearing electron-withdrawing (i.e., NO_2_, Cl) and -donating (i.e., OH) groups, were brominated in good to excellent yields (75–95%). Based on the experimental data, both TBHP and H_2_O_2_ had similar bromination efficiency. Ring bromination instead of α-bromination exclusively occurred for OH-substituted ketones, highlighting this routes’ selectivity issues.

**Scheme 37:**
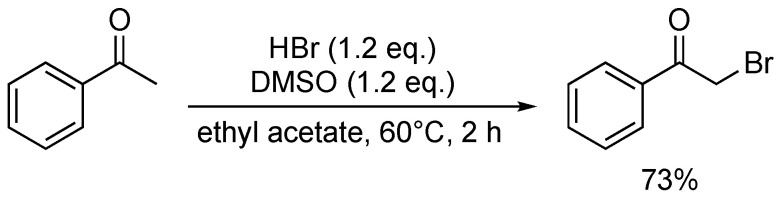
DMSO- and HBr-mediated bromination of acetophenone.

DMSO was also shown to be an effective oxidant in the HBr-mediated bromination of ketones (Scheme 37) [100]. However, the substrate scope was again limited, making it difficult to evaluate the system’s efficacy and overall applicability.

An alternative HBr-based bromination was reported by Li and co-workers [101], which made use of Cu(NO_3_)_2_ (0.5 equiv.) and molecular oxygen to mediate the halogenation. Although the scope is not very broad, the bromination yields were quite good (83–94%). In most cases, dibromination of the substrate was observed, which again complicates purification.

**Scheme 38:**
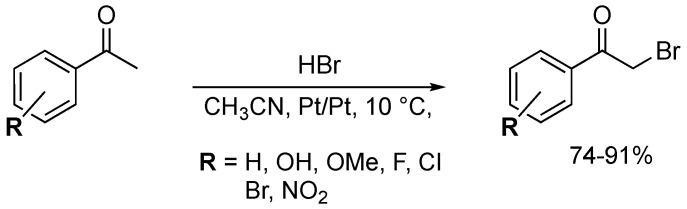
Electrochemical HBr-mediated direct bromination.

The electrochemical α-bromination of aryl ketones was also reported by Kulandainathan and co-workers [102]. HBr was added to the ketone in acetonitrile in an undivided electrolytic cell containing two Pt electrodes (Scheme 38). A variety of aryl ketones were brominated in good to excellent yields. This method’s fine control over the experimental conditions through its set-up is quite intriguing. Despite the acidic conditions, the presence of acid-sensitive functional groups (i.e., OH) was well tolerated. In addition, this route showed excellent selectivity, as only monobrominated products were observed.

**Scheme 39:**
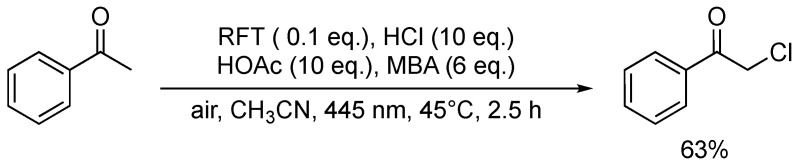
Photocatalysed direct halogenation of acetophenone.

In 2008, a photocatalytic flavin-based method was developed for the chlorination of arenes and aromatic ketones [103]. Halogenases, enzymes catalysing the chlorination of aromatic compounds, provided the inspiration for this catalytic system. Stirring a solution of riboflavin tetraacetate (RFT), methoxy benzyl alcohol (MBA) and HCl in acetonitrile under visible light irradiation, afforded a range of chlorinated arenes. α-chlorination occurred when phenylethanone was used, generating the desired α-bromoketone (Scheme 39). The authors claimed that, under these conditions, the peroxide concentration was kept consistently low, preventing undesired polychlorination of the substrates. Despite the moderate chlorination yields, this route stands out as it is the only catalytic direct halogenation route to date, that mimics an enzymatic process. 

**Scheme 40:**
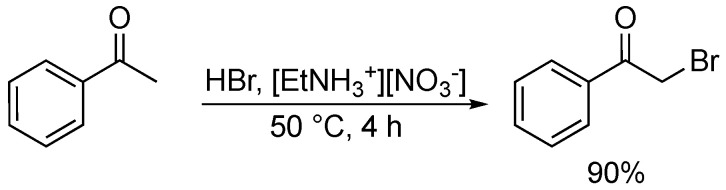
[EtNH_3_^+^][NO_3_^−^]/HX-mediated bromination of 1-phentylethanone.

ILs are generally considered better alternatives to typical organic solvents as reaction media [104], mainly due to their recyclability and the subsequent ease in product isolation, as biphasic systems can be readily designed. In 2013, Stavber and Laali developed an ethylammonium nitrate ([EtNH_3_^+^][NO_3_^−^])- and HX-mediated bromination and chlorination, yielding a variety of ring halogenated arenes. Under these conditions, the α-bromoketone was exclusively obtained in 90% yield upon addition of HBr and [EtNH_3_^+^][NO_3_^−^] to 1-phentylethanone (Scheme 40). However, the recurring problem of ring bromination was observed for the *p*-OMe substituted phenylethanone.

**Figure 6:**
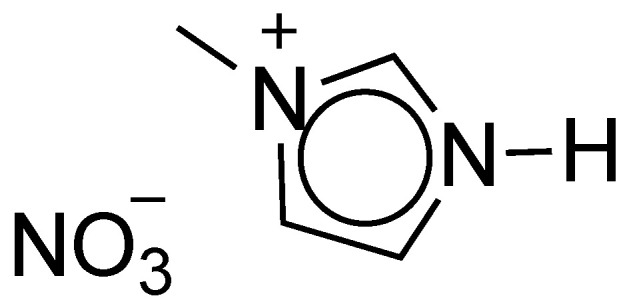
[Hmim][NO_3_], as co-solvent and promotor of the direct halogenation.

Another ring halogenation carried out in an IL was reported by Chiappe and co-workers [105]. Herein, the role of 3-methylimidazolium nitrate ([Hmim][NO_3_]) was shown to be twofold; it acts as a co-solvent and promotor of the halogenation (Figure 6). At this point, it is widely believed that the use of ILs in these reactions will always involve some sort of IL-based activation role. Under these conditions, aryl ketones showed exclusive α-chlorination. 2-Bromo-1-phenylethanone was isolated in 90% yield when the IL (2 equiv.) and an aqueous HCl solution (1 equiv.) were added to the corresponding ketone. Recycling experiments demonstrated that the IL could be reused in four successive runs without a significant loss in activity. The scope was limited to one aryl ketone, which again makes it difficult to assess this route’s applicability.

#### 2.1.5. Polymeric Halogenation Agents

**Figure 7:**
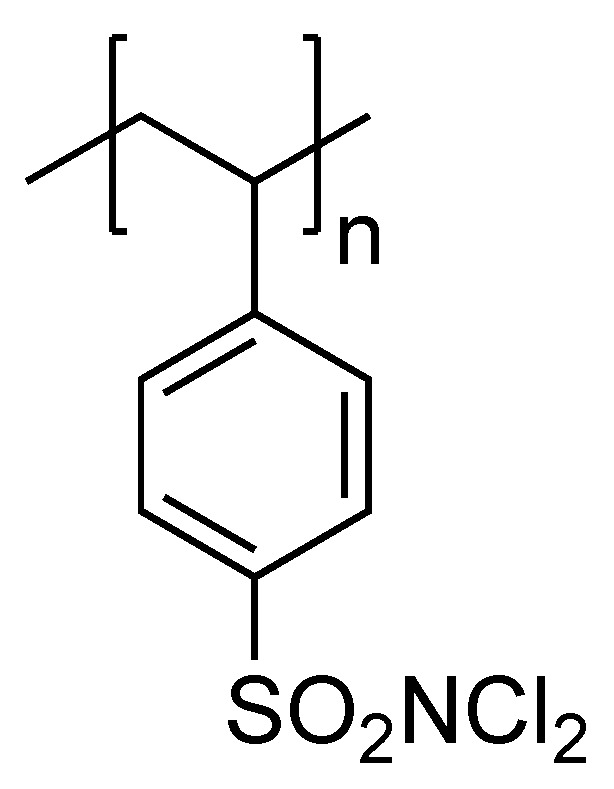
Poly(4-vinyl-*N*,*N*-dichlorobenzenesulfonamide) as an effective chlorination agent.

Poly(4-vinyl-*N*,*N*-dichlorobenzenesulfonamide) was first used for the halogenation of one aryl ketone, yielding 2-chloro-1-phenylethanone in a moderate 40% yield (Figure 7) [106]. The straightforward reaction conditions and recyclability of the chlorination agent are the main advantages of the protocol. However, prior to halogenation, the ketone deprotonation had to be performed separately using NaH.

**Scheme 41:**
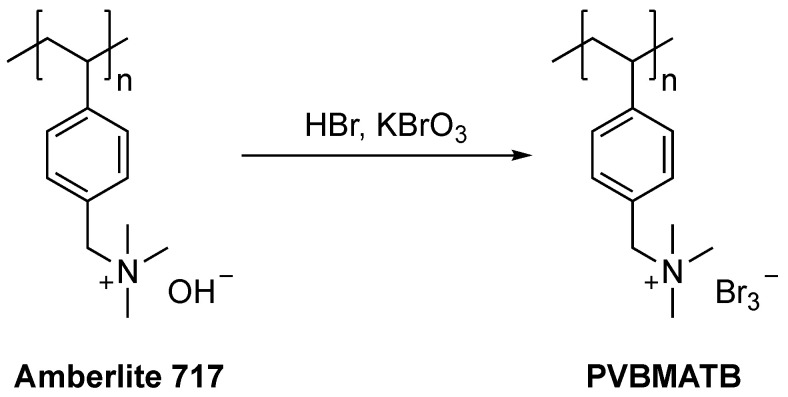
Synthesis of the PVBMATB resin.

In 2019, a simple and green bromination method was developed by Han and co-workers [107], whereby poly(vinylphenyltrimethylammonium tribromide) (PVBMATB) was synthesised by treating the Amberlite 717 resin with HBr and KBrO_3_ (Scheme 41). The resin proved to be an effective brominating agent as it accommodated the halogenation of eleven aryl ketones in moderate to excellent yields (60–96%). *Ortho*-substituted ketones were less efficiently brominated, highlighting this route’s limitations with sterically hindered substrates. PVBMATB provided a visual feedback system for the reaction progress, as its colour changed from red to gold when the reaction is completed. The authors claimed that the resin could be recycled and reused up to three consecutive runs with no significant loss in activity. This route is highly valuable from both economic and environmental points of view, associated with the low cost and recyclability of PVBMATB.

**Figure 8:**
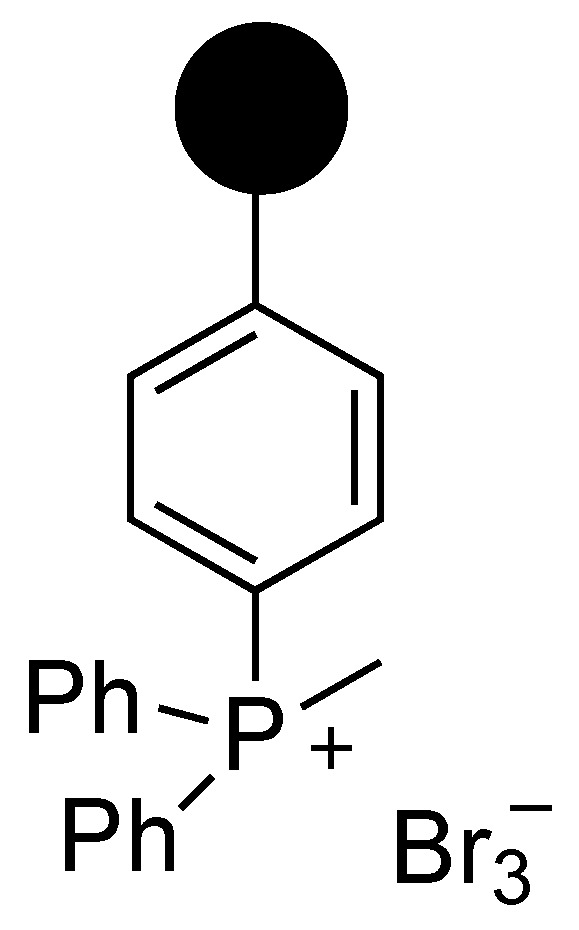
Methyl-triphenylphosphonium bromide supported on polystyrene.

Weiss and others showed that a resin, obtained upon treatment of diphenylphosphine-functionalised polystyrene with CH_3_Br and Br_2_, exhibited an excellent bromination activity (Figure 8) [108]. The bromination of a variety of alkenes, alkynes and ketones was carried out in DCM, a swelling solvent for the polymeric agent. Only one aromatic ketone was halogenated via this protocol, as 2-bromo-1-phenylethanone was obtained in 98% yield. The α,α-dibromination was always observed, regardless of the nature of the substrate. For comparison, the authors designed a homogenous methyl-triphenylphosphonium bromide-catalysed halogenation in DCM which yielded 2-bromo-1-phenylethanone in 80% yield, which is lower than the heterogeneous conditions used above. This route’s main features are its simple work-up and product isolation, in conjunction with the recyclability of the bromination agent.

**Scheme 42:**
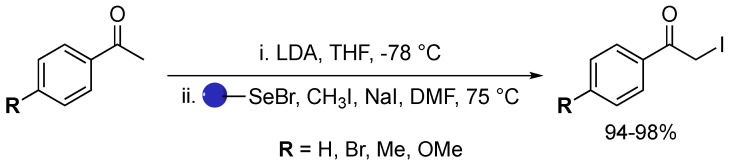
Liu’s polymer-supported iodination.

Liu and co-workers reported a polymer-supported halogenation agent which mediated the selective α-iodination of four aromatic ketones in excellent yields (Scheme 42) [109]. The polystyrene-bound selenium bromide was regenerated upon treatment with Br_2_. However, lithium diisopropylamide (LDA) had to be employed prior to halogenation to generate the nucleophilic enolate, which is not ideal, especially when other straightforward one-step options are available. 

#### 2.1.6. Ionic Liquids as Halogenation Agent

**Figure 9:**
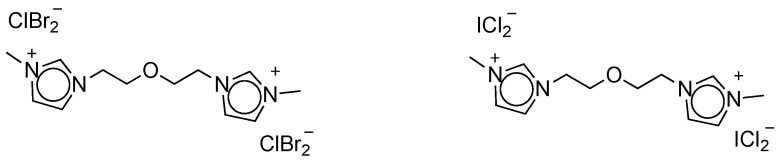
Effective IL-based halogenation agents.

IL-based halogenation agents are gaining traction in the literature nowadays, mainly due to their contribution to the good practice ethos in terms of recyclability and reusability. In 2015, α-bromination and α-chlorination procedures mediated by, respectively, bis(2-*N*-methylimidazoliumethyl)-ether dibromochlorate and dichloroiodate, were reported (Figure 9) [110]. Eight α-bromoaryl ketones were isolated in excellent yields (88–95%), upon the addition of bis(2-*N*-methylimidazoliumethyl)-ether dibromochlorate (0.5 equiv.) to the corresponding ketones under reflux for 8–24 h. Similarly, the reaction with the dichloroiodate analogue generated eight α-chloroketones in 88–93% isolated yield. This procedure tolerated the presence of electron-withdrawing (i.e., Br, Cl, NO_2_), -donating (i.e., Me, OMe) and acid-sensitive (i.e., OH) groups on the substrates, highlighting its wide applicability. The authors claimed that the ILs can be regenerated and reused; however, no experimental details were provided to support this claim.

**Figure 10:**
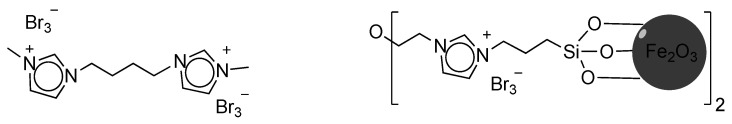
Bis(imidazolium)-based (**left**) and Fe_2_O_3_-supported (**right**) ditribromide ILs as effective halogenation agents.

Veisi and Sedrpoushan illustrated that 1,4-bis(3-methylimidazolium-1-yl)butane ditribromide was an effective bromination agent for arenes and aryl ketones (Figure 10, left) [111]. Upon addition of the IL (0.5 equiv.) in CH_3_CN, α-bromination of aryl ketones was achieved, yielding three α-bromoketones (i.e., *p*-H, -Me, -OMe) in excellent yields (92–96%). The authors demonstrated that the IL could be regenerated and reused for five successive cycles without a significant loss in activity. The straightforward isolation of the brominated products further highlighted this route’s sustainable character (i.e., adding water to the reaction mixture).

Another IL-based brominating agent, immobilised on Fe_2_O_3_ support, was successfully utilised for the bromination of a variety of alkenes, alkynes, arenes and aryl ketones (Figure 10, right) [112]. The bromination agent was easily separated from the reaction mixture by using an external magnet. When recovered, the authors claimed that the reagent could be reused up to six consecutive runs without any significant loss in activity. Four α-brominated aromatic ketones were isolated in good to excellent yields (80–90%). The presence of electron-donating substituents decreased the reaction time from eight (for phenylethanone) to six hours (for the *p*-OMe substituted analogue). Aryl ketones bearing electron-withdrawing groups (i.e., *p*-Cl and *o*-NO_2_) showed a similar trend.

**Scheme 43:**
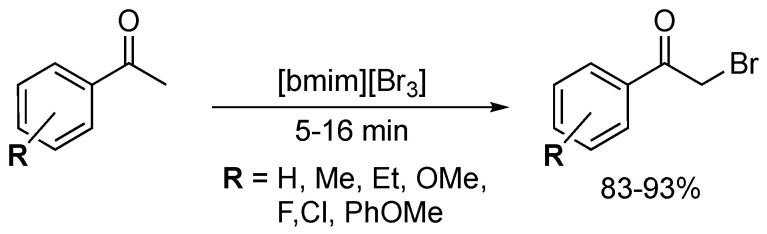
Coa and Hu’s α-bromination procedure.

An efficient IL-mediated bromination protocol was also developed by Coa and Hu [113]. [Bmim][Br_3_] mediated the bromination of six aromatic ketones in good to excellent yields (Scheme 43). The procedure tolerated the presence of both electron-donating and -withdrawing substituents on the phenyl ring. The authors stated that the IL could be recycled several times without any loss in bromination efficacy; however, no experimental data were provided in support of this claim. The short reaction times were an important feature of this IL-based halogenation.

**Figure 11:**
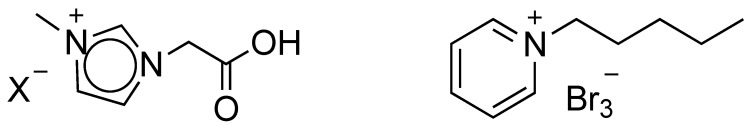
[Acmim]X (**left**) and pentylpyridinium tribromide (**right**).

In 2007, Ranu and co-workers designed an acetylmethylimidazolium halide ([Acmim]X) (X = Cl^−^, Br^−^) and ceric ammonium nitrate-based α-chlorination and α-bromination of ketones (Figure 11, left) [114,115]. Addition of the IL (1.2 equiv.) and ceric ammonium nitrate (CAN) (2 equiv.) to an aryl ketone, yielded three α-chloro and three α-bromoketones (i.e., *p*-H, -Me and -OMe substituted) in good yields (80–82% and 80–85%, respectively). The authors mentioned that the selective α-halogenation of this protocol is linked to its radical nature.

Alternatively, a solvent-free protocol mediated by pentyl-pyridinium tribromide was designed and reported by Dorta and Salazar (Figure 11, right) [116]. Only one aryl ketone was tested, as only 2-bromo-1-phenylethanone was isolated in 85% yield. Furthermore, the neat conditions were offset by using ether for extraction purposes during the work-up. The authors stated that the tribromide reagent could easily be regenerated from the reaction mixture using Br_2_.

**Figure 12:**
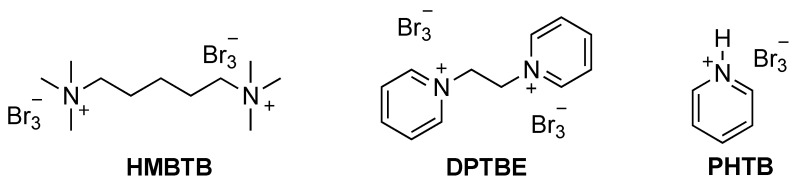
Other tribromide-based halogenation agents.

Patel and co-workers designed another recyclable bromination agent, i.e., hexamethonium bis(tribromide) (HMBTB) (Figure 12, left) [117]. The procedure involved grinding a solid mixture of HMBTB (0.5 equiv.) and ketones in a mortar for 20–60 min at room temperature. The method demonstrated a broad substrate scope, with aromatic ketones showing exclusive α-bromination. Similarly, the solvent-free α-bromination was also mediated by 1,2-dipyridiniumditri-bromide-ethane (DPTBE) (Figure 12, middle) [118]. DPTBE (0.5 equiv.) was added to the aromatic ketone, and subsequent grinding of the solids in a mortar at room temperature for 1–1.5 h yielded the desired product. This route tolerated the presence of electron-withdrawing (i.e., Cl, NO_2_) and electron-donating groups (i.e., OMe, OCH_2_Ph). Five *para*-substituted α-bromoketones were isolated in good to excellent yields (82–89%). Other tribromides, such as pyridinium hydrotribromide (PHTB), also showed excellent bromination activity (Figure 12, right). PHTB mediated the bromination of several *para*- and *meta*-substituted aromatic ketones (i.e., -OMe, Cl, NO_2_, Br) in good to excellent yields (70–98%) [119].

#### 2.1.7. Miscellaneous Halogenation Agents

Other halogenation strategies include the use of transition metal halides. Sun demonstrated the halogenation activity of copper halides in the direct halogenation of ketones [120]. Addition of Ti-Al binary oxides (0.02 equiv.) and CuX_2_ (2 equiv.) to phenylethanone, yielded the corresponding α-bromo and α-chloroketones in 42 and 56% isolated yield, respectively. The bromination reaction was carried out in acetonitrile, while the chlorination required formic acid as solvent. For both, the α,α-dihalogenated species were observed as an important side product (isolated in 20% and 10% yield, respectively), highlighting this route’s moderate selectivity. 

**Scheme 44:**
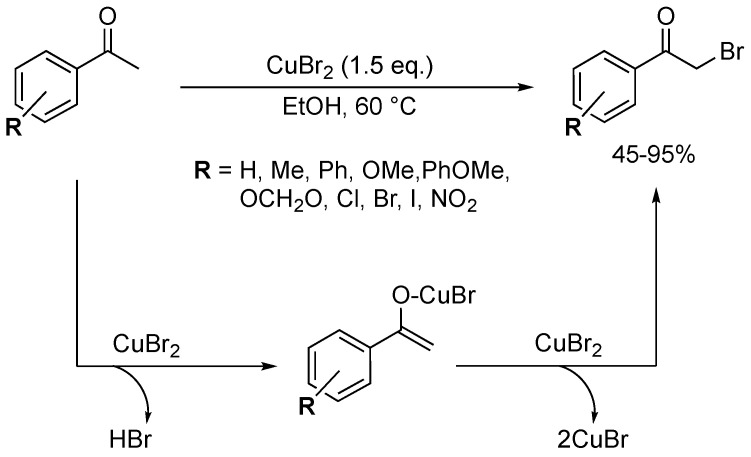
Straightforward CuBr_2_-based halogenation.

In contrast to Sun’s route, a broader scope of α-bromoketones were reported by Cai, Peng and An [121,122]. Eleven ketones were brominated upon the addition of 1.5 equiv. of CuBr_2_ (Scheme 44). At first sight, the mechanism seemed to indicate that at least 2 equiv. of CuBr_2_ are needed to achieve full conversion; however, the first step involving the formation of the copper enolate could occur with CuBr instead. Additionally, CuBr in the presence of HBr can disproportionate back to CuBr_2_, thus regenerating the active species. The procedure tolerated the presence of electron-donating and -withdrawing groups on the aryl substrates. NO_2_-substituted aromatic ketones underwent a less efficient halogenation compared to the unsubstituted aryl ketone. Nevertheless, other electron-poor substrates (i.e., bearing Cl, Br, I substituents) did not exhibit such a decrease in bromination efficiency. An inherent drawback of these CuX_2_-mediated reactions is the moderate halogen atom economy.

Another transition metal-based protocol for the halogenation of ketones was reported by Zhang and co-workers using FeCl_3_·6H_2_O and phenyliodinium diacetate in HOAc [123]. Addition of iron chloride (2 equiv.) and oxidant (1.2 equiv.) to the substrate, yielded 25 α-chlorinated aryl ketones in moderate to good yields. The acidic reaction conditions limited the substrate scope, as it decreased this route’s functional group tolerance (e.g., NH_x_, OH).

**Scheme 45:**
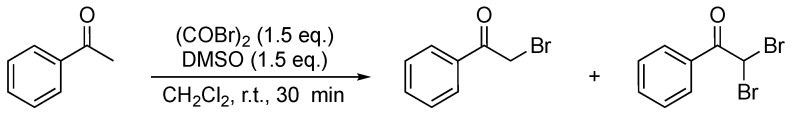
DMSO- and (COBr)_2_-mediated bromination.

Tian and Sun developed a DMSO and oxalyl bromide ((COBr)_2_)-based bromination method for a diverse set of alkenes, alkynes and ketones (Scheme 45) [124]. The procedure leads to moderate selectivity, as the α-brominated and α,α-dibrominated products were obtained in a 1:1 ratio. Despite the relatively mild reaction conditions and short reaction times, this route does require further optimisation for more selective α-bromination. 

**Scheme 46:**
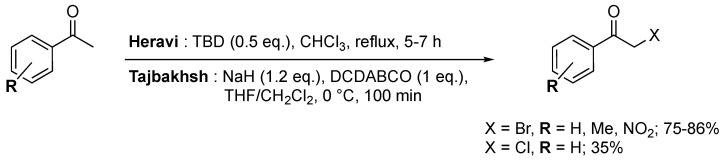
DABCO-mediated halogenations.

Both Tajbakhsh and Heravi illustrated that 1,4-diazabicyclo[2,2,2]octane (DABCO) can be employed as a halogenation agent (Scheme 46) [125,126]. Heravi and co-workers used tetrameric DABCO-bromine (TBD) supported on silica or alumina to efficiently mediate the α-bromination of four aromatic ketones in good yields. The non-hygroscopic supported solid performed the bromination under mild reaction conditions with an easy work-up (simple filtration). Tajbakhsh and co-workers, on the other hand, employed DABCO as a chlorination agent after treatment with chlorine gas, affording 1,4-dichloro-1,4-diazabicyclo[2,2,2]octane (DCDABCO) (Scheme 46). A set of alkenes, alkynes and ketones were chlorinated via this method, among which stands one, aryl ketone. The authors claim that DCDABCO can be reused for four consecutive reactions without significant loss in activity.

**Figure 13:**
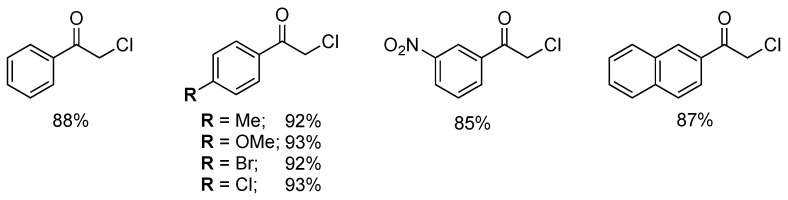
Scope of the SiCl_4_- and UHP-mediated bromination.

**Scheme 47:**
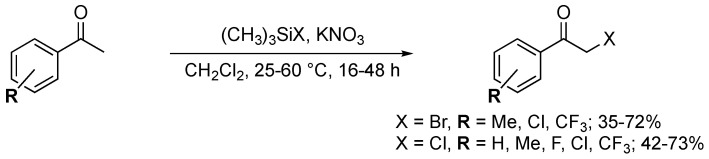
Halogenation protocol by Prakash and Mathew.

SiCl_4_ was also shown to be an excellent chlorinating reagent. In 2010, El-Ahl illustrated that a SiCl_4_ and UHP/iodosylbenzene-based system mediated the chlorination of a broad range of ketones in good to excellent yields (Scheme 47, Figure 13) [127]. Aryl ketones underwent exclusive α-bromination, highlighting the excellent regioselectivity of this route.

An alternative versatile α-halogenation method was reported by Prakash and Mathew in 2011 [128]. An effective halogenation agent was obtained upon the coupling of bromo- or chlorotrimethylsilane with KNO_3_. The procedure yielded eight α-chloroketones and five α-bromoketones (Scheme 47). Dihalogenation was a burdensome issue in this Si-mediated reaction. The α,α-dihalogenated products were obtained for each substrate, complicating purification.

**Figure 14:**
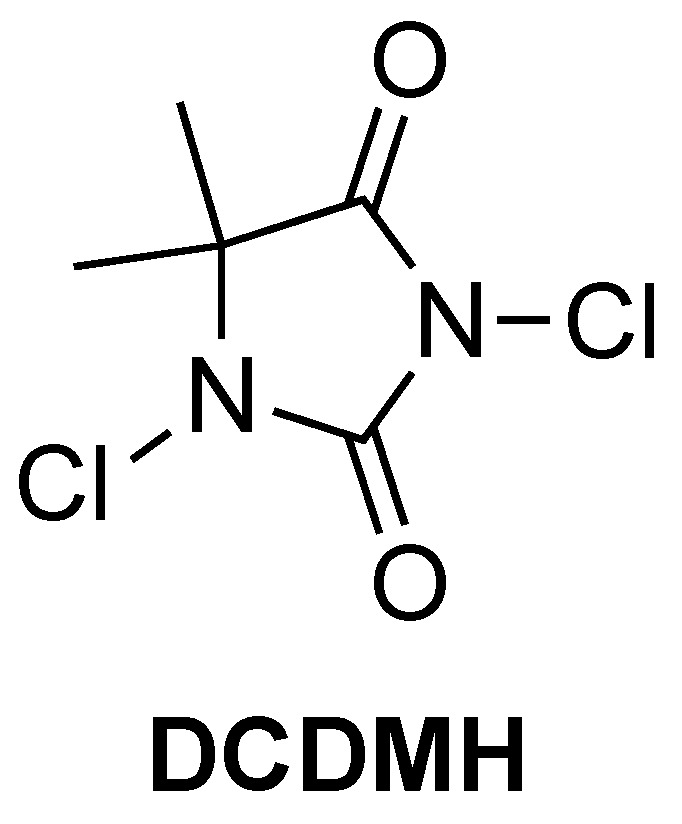
Alternative halogenation agents.

Zou demonstrated that in the α-chlorination of aromatic ketones, 1,3-dichloro-5,5-dimethyl-hydantoin (DCDMH) acts as an inexpensive chlorination agent (Figure 14) [129]. A silica gel-supported route and a *p*TsOH-catalysed method were therefore developed. The former converted the aromatic ketones with a significantly higher efficiency than the latter. Together with the shorter reaction times (1 h vs. 8 h, respectively), the SiO_2_-supported route seemed to be superior. However, this route was not as selective as the *p*TsOH promoted version. The chlorination of nitro-substituted aromatic ketones yielded a mixture of the α-chlorinated ketone and the ketal. Furthermore, for methoxy-substituted substrates, ring chlorination was exclusively observed.

Alumina-supported α-bromination and α-iodination protocols have been developed [130]. Gupta described a solvent-free hexamethylenetetramine-bromine (HMTAB)-mediated halogenation. Eleven aromatic α-bromoketones were obtained in good yields (70–80%) upon grinding HMTAB (1.6 equiv.), basic alumina and the substrate in a mortar. The homogenous solid mixture was subsequently irradiated in a MW for 5–10 min. Electron-withdrawing and -donating groups were well tolerated, as well as acid-sensitive ones (e.g., OH, NH_x_). The main advantages of this procedure are its short reaction times and the solvent-free conditions.

CAN demonstrated excellent catalytic activity in the α-chlorination of (a)cylic ketones [131,132]. This route employed the rather unconventional acetyl chloride as the chlorine source, yielding one α-chloroarylketone (i.e., 2-chloro-1-phenylethanone) in 87%. The authors claimed that α-chlorination was exclusively observed.

**Scheme 48:**
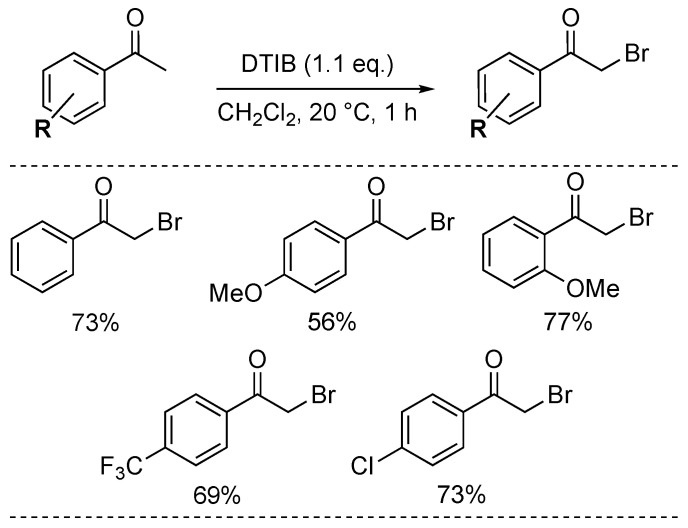
DTIB-mediated bromination of aromatic ketones.

In 2019, Matsubara and co-workers designed a bis(1,3-dimethyl-2-imidazolidinone)hydrotribromide (DITB)-mediated halogenation [133]. The α-bromination of a set of ketones was established upon the addition of DTIB (1.1 equiv.). Seven α-bromoketones could be isolated among which five aromatic examples could be isolated in moderate to good yields (Scheme 48). Methoxy-substituted aryl ketones were obtained in significantly lower yields, which was ascribed to dibromination of that substrate.

### 2.2. Nucleophilic Halogenation

The nucleophilic halogenation approach to generate α-halogenated ketones is much less attractive than its electrophilic counterpart, mainly due to the need for an intermediary step to install a leaving group on the carbon atom of the enolate. This in turn generates unnecessary waste. Nonetheless, in this section, we would like to mention a few reports to better clarify this approach.

**Scheme 49:**
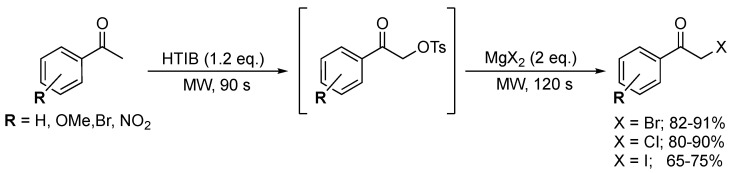
MgX_2_- and HTIB-based halogenation of ketones.

A clear example of this strategy can be found in the report by Lee and co-workers describing the solvent-free MgX_2_ and HTIB-based halogenation (Scheme 49) [134]. The generality of this route was demonstrated by the bromination, chlorination and iodination of a set of aryl ketones under the same reaction conditions. Phenylethanone derivatives were brominated, chlorinated, and iodinated in good to excellent yields (82–91%, 80–90% and 65–75%, respectively). The procedure was thus very effective for both the bromination and chlorination; however, a slight decrease in efficiency was observed for the iodination. This route tolerated the presence of both electron-withdrawing (i.e., Br, NO_2_) and -donating groups (i.e., OMe) on the substrates. The reaction is performed in two steps; the first one using HTIB installs an OT leaving group on the α-carbon atom, and the following step consists of Br^−^ nucleophilic substitution on the same carbon to afford the corresponding α-halogenated ketone.

**Scheme 50:**
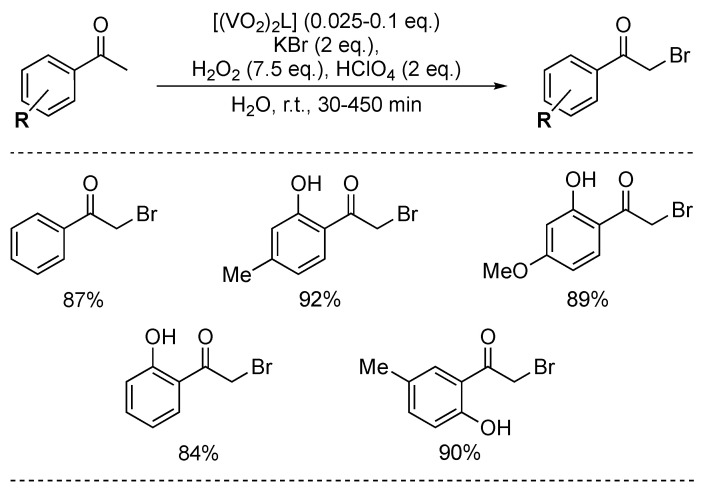
V(V)-catalysed bromination.

In an attempt to circumvent the need for stoichiometric amounts of a leaving group, Lal and co-workers designed and reported a dioxidevanadium(V)-catalysed bromination (Scheme 50) [135]. Five ketones were brominated in good to excellent yields. Remarkably, the procedure tolerated acid-sensitive substituents (i.e., OH) on the aromatic ring. However, large amounts of H_2_O_2_ as oxidant were needed. The authors gave a plausible mechanism, involving the initial formation of vanadium C-enolate via C-H activation of the ketone, followed by a Br^−^ nucleophilic attack on the same carbon, which liberates the vanadium complex and generates the α-bromoketone, thus closing the catalytic cycle. However, in our opinion, an electrophilic halogenation mechanism could not be excluded, since the protocol operates under harsh oxidative conditions; H_2_O_2_ or even the vanadium catalyst can readily oxidise Br^−^ into Br_2_ (or HOBr after reaction with water), which would then be capable of reacting directly with the ketone.

## 3. Oxyhalogenation of Hydrocarbons

In the last two decades, alternative protocols have been developed that have targeted the numerous issues raised by the direct halogenation approach. A vast amount of work has been dedicated to the oxyhalogenation of alkenes, alkynes, and secondary alcohols. Nonetheless, these routes either only partially tackled the problems associated with the direct halogenation (e.g., oxidation often required an acidic promotor), or present moderate efficacy and/or selectivity. Herein, we showcase some of the most attractive strategies.

### 3.1. Alkenes as Substrates

**Scheme 51:**
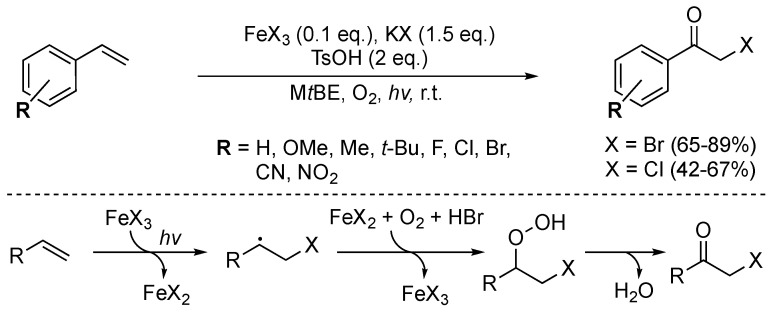
FeX_3_- and KX-mediated oxyhalogenation of styrenes.

Styrene derivatives have proven to be excellent oxyhalogenation substrates. In 2020, Zhu and co-workers developed a FeX_3_-catalysed oxyhalogenation mediated by KX in methyl *tert*-butyl ether (MTBE) (Scheme 51) [136]. Fourteen α-bromo and fourteen α-chloroketones were isolated in moderate to good yields. Electron-poor, -rich and sterically hindered substrates were efficiently functionalised by the developed procedure. However, the protocol failed in the oxyhalogenation of *p*-OH substituted alkenes, attributed to the use of TsOH. The reaction proceeds via a radical mechanism, where the Br radical is trapped by the styrene derivative. This radical intermediate is then captured by O_2_ and FeBr_2_, affording a peroxide intermediate which upon water elimination yielded the corresponding halogenated ketone. Additionally, the oxyhalogenation presents a substrate specific character, as the oxychlorination yields were significantly lower than the oxybromination relatives.

**Scheme 52:**
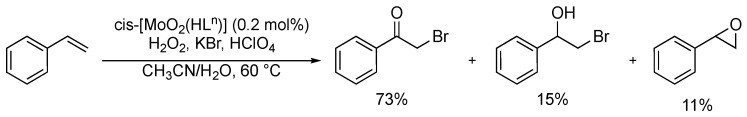
Molybdenum(VI)-catalysed oxybromination of olefins.

Another metal-catalysed oxybromination of olefins was established using a molybdenum(VI)-based complex (Scheme 52) [137]. 2-Bromo-1-phenylethanone was obtained in 73% yield upon addition of 0.2 mol% of a cis-dioxomolybdenum(VI) complex to a mixture of H_2_O_2_, KBr, HClO_4_ and styrene. The bromohydrin and epoxide were isolated as important side products (in 15 and 11%, respectively), highlighting the moderate catalyst selectivity.

**Scheme 53:**
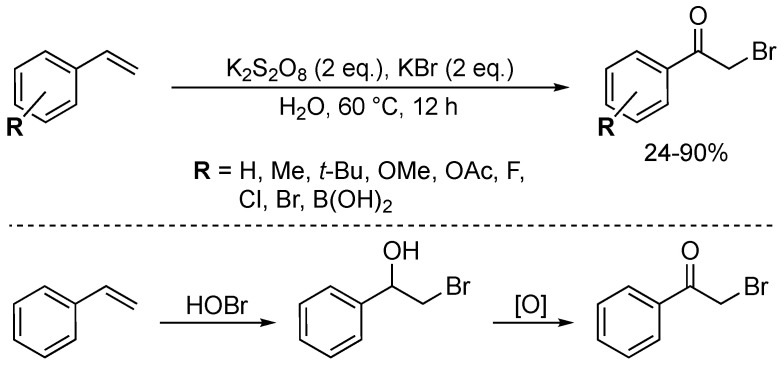
Oxybromination of styrene derivatives by Guo and co-workers.

Guo and co-workers reported the oxybromination of 11 styrene derivatives upon the addition of KBr (2 equiv.), K_2_S_2_O_8_ (2.5 equiv.) in H_2_O (Scheme 53) [138]. After stirring for 12 h at 60 °C, the corresponding aryl bromoketones were isolated in low to excellent yields (24–90%). The method tolerated substrates bearing electron-donating (e.g., Me, *t*-Bu) or -withdrawing (e.g., F, Cl, Br) substituents on the aromatic ring. A significant decrease in hydration efficiency (i.e., 24%) was observed for the OMe-substituted styrene derivative. Furthermore, this route was not compatible with heteroaromatic substrates. Despite its shortcomings, the developed procedure did benefit from readily available starting materials and a safe halogen source. The mechanism involves the initial K_2_S_2_O_8_-mediated oxidation of Br^−^ to Br_2_, which leads to the formation of HOBr in the presence of water. Addition of HOBr onto the alkene affords the bromohydrin intermediate. Upon oxidation of the latter, the corresponding bromoketone is obtained. In 2019, the authors slightly altered their oxyhalogenation procedure [139]. A KI- and TBHP-based system transformed styrene into the corresponding α-iodoketone in 97% yield.

Adimurthy and Iskra independently established an aqueous H_2_O_2_-based oxyhalogenation method [140,141]. Both authors reported the use of HBr as a bromination agent, which generated the corresponding α-bromoketone and the bromohydrin (Table 2). Iskra and co-workers demonstrated that using NBS instead of HBr considerably shifted the selectivity towards the α-haloketone. Despite enhanced selectivity, the hydrin intermediate was still observed in 19% yield. Furthermore, the reported substrate scope consisted of only styrene. The α-haloketone selectivity appeared to be less problematic in the Adimurthy route (Table 2), and a broader scope was established; eight aromatic α-bromoketones were isolated in low to excellent yields (20–94%). Although electron-donating aromatic substituents (e.g., Me, *t*-Bu) were well tolerated, electron-withdrawing substituents (e.g., Cl, Br, NO_2_) were not. Moreover, the presence of steric bulk close to the alkene centre appeared to have a detrimental effect on the system’s efficiency.

**Scheme 54:**
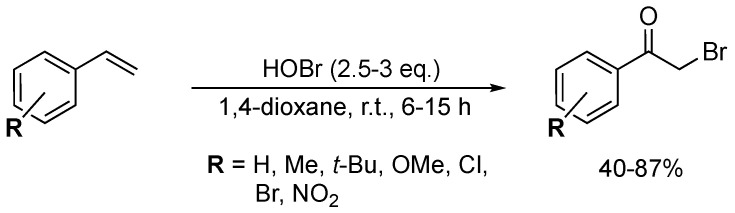
The use of HOBr in the oxybromination of alkenes.

Adimurthy and Ranu discussed the use of HOBr in the oxybromination of alkenes (Scheme 54) [142]. Their procedure involved the addition of two equivalents of HOBr to various olefins in 1,4-dioxane. Several α-bromoketones were obtained in moderate to excellent yields (40–87%). Regardless of the electronic properties of the substrate, the bromohydrin analogue was consistently observed in significant amounts.

**Scheme 55:**
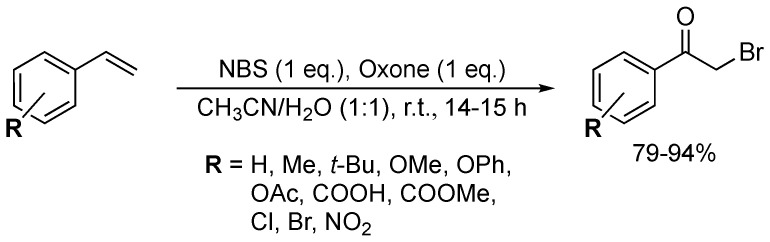
NBS/Oxone system for the oxyhalogenation of olefins by Moorthy and co-workers.

Moorthy and co-workers designed a NXS oxyhalogenation of olefins in DMSO, which employed 2-iodoxybenzoic acid (IBX) as the oxidant [143]. More recent work involved the in situ generation of an IBX derivative via Oxone^®^ (Scheme 55) [144]. This versatile method generated a set of *para*- and *ortho*-substituted α-bromoketones in good to excellent yields (79–94%). Electron-withdrawing and -donating groups on the substrates were well tolerated by the NBS and Oxone^®^ system. Meanwhile, Mal and co-workers demonstrated the solvent-free oxyhalogenation of a variety of alkenes using a NXS (1.1 equiv.) and IBX (2 equiv.) system [145]. The procedure generated 2-bromo- and 2-iodo-1-phenyl-ethanone in, respectively, 86% and 89% yield. The authors claimed that the obtained ketones did not require chromatographic purification, facilitating product isolation significantly. They further stated that the oxidant could be recycled via its waste product 2-iodosobenzoic acid. However, product loss was observed and attributed to the high volatility of the halogenated products.

**Scheme 56:**
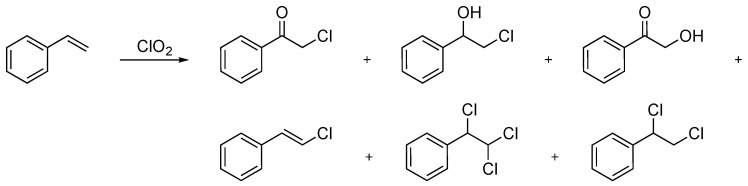
Selectivity issues of the ClO_2_-mediated oxychlorination.

Phukan and co-workers designed an effective *N*,*N*-dibromo-*p*-toluenesulfonamide (TsNBr_2_)-mediated transformation of alkenes into their corresponding α-bromoketones [146]. Both electron-poor and -rich styrene derivatives were efficiently converted when adding TsNBr_2_ (2.2 equiv.) to the starting material in an acetone/H_2_O mixture at room temperature. The resulting products were isolated in good to excellent yields (80–87%). This route stood out due to its mild reaction conditions and good efficiency, although it should be noted that TsNBr_2_ is not readily accessed [147,148]. In 2017, Loginova et al. reported a ClO_2_-mediated oxidative chlorination of styrene [149]. This route displayed poor selectivity issues as next to the desired α-chloroketone, five side-products were obtained, significantly complicating the product isolation process (Scheme 56). Furthermore, chlorine dioxide is an extremely toxic and dangerous reagent; its use thus requires strict safety precautions.

**Scheme 57:**
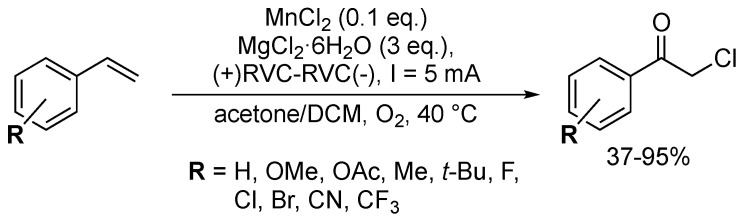
Chen’s electrochemical oxychlorination.

Chen and co-workers designed an electrochemical Mn-catalysed oxychlorination procedure, using a reticulated vitreous carbon (RVC)-based anode and cathode (Scheme 57) [150]. Fourteen electron-poor and -rich α-chloroketones were isolated in low to excellent yields. The developed route was not compatible with sterically hindered substrates, as the *ortho*-substituted product was obtained in only 37% yield. As the FeBr_3_-based protocol, the role of a peroxide intermediate was noted.

Itoh and co-workers developed an aerobic photo-oxidative protocol yielding α-haloketones from their styrene derivatives [151]. The reaction conditions and scope are depicted in Table 3.

**Scheme 58:**
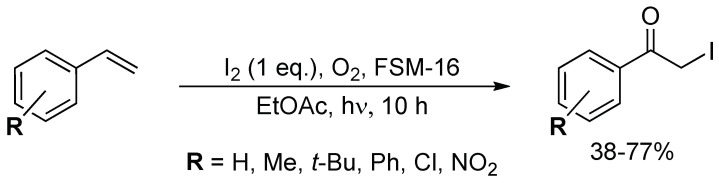
Itoh’s procedure using irradiation.

In 2007, Itoh described the synthesis of α-iodoketones upon irradiation with a fluorescent lamp of a mixture of I_2_ (1 equiv.), O_2_ and FSM-16 (mesoporous silica) in EtOAc; with FSM-16 acting as a heterogeneous catalyst (Scheme 58) [152]. Several α-iodoketones were obtained from their styrene derivatives in moderate to good yields (38–77%). The experimental data showed that the system coped well with the presence of electron-withdrawing (e.g., Cl, NO_2_) and -donating (e.g., Me, *t*-Bu) substituents on the aromatic ring. However, this route was incompatible with sterically hindered alkenes.

**Scheme 59:**
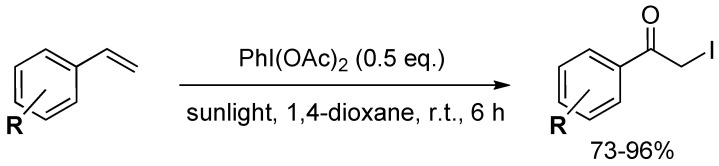
Zhang’s versatile oxyiodination.

Zhang and co-workers utilised 1,3-dibromo-5,5-dimethylhydantoin (DBH) for the oxybromination of styrene derivatives in H_2_O [153]. The role of DBH (2 equiv.) was twofold, since it acted as an oxidant and as halogenation agent. This protocol yielded 11 aromatic α-bromoketones bearing a diverse set of aromatic substituents (34–85%). This route was not compatible with sterically hindered alkene centres, as the *o*-NO_2_ substituted ketone was isolated in only 34% yield. In 2020, Wang and Zhang reported an elaborate scope (18 examples) of α-iodoketones, obtained in good to excellent yields (73–96%), from corresponding olefins [154]. The reaction was catalysed by visible light under mild experimental conditions (Scheme 59).

### 3.2. Alkynes as Substrates

**Scheme 60:**
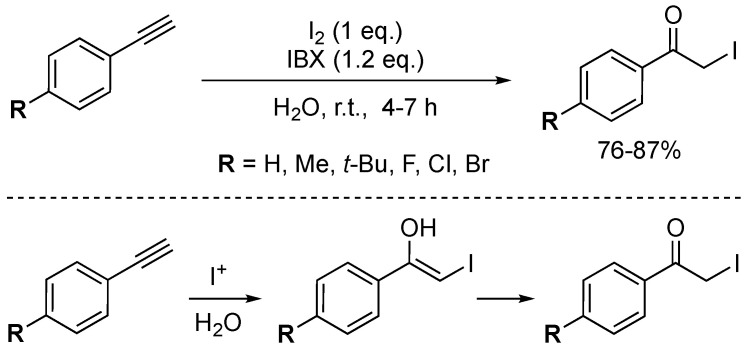
I_2_- and IBX-based oxyiodination of alkynes.

Similarly to the alkene systems mentioned above, I_2_- and IBX-based oxyiodination of aromatics was also applied to alkynes in water (Scheme 60) [155]. The protocol showed good tolerance towards electron-donating and electron-withdrawing substitutions on the aromatic alkynes. The role of IBX is to facilitate the initial addition step of I^+^ and water to alkyne, most likely to generate the I^+^ active species from I_2_. Afterwards, the formed iodo-enol compound would tautomerise to the iodoketone form, affording the target compound.

Ahmed and co-workers described an alternative I_2_-based oxyhalogenation protocol which generated 11 α-chloroketones in good yields (69–79%) upon the addition of HCl to the corresponding phenylacetylene derivatives in DMSO [156]. The protocol tolerated the presence of electron-withdrawing (i.e., F, Br, Cl, CF_3_) and -donating (i.e., Me, *n*-Bu) aromatic substituents. Interestingly, the presence of steric bulk near the alkyne centre (ortho-substitution of the aromatic ring) did not affect its efficacy. Acid-sensitive substrates were not tolerated under these conditions, thus limiting the reaction scope.

Phukan and co-workers reported the oxybromination of aromatic alkynes mediated by a TsNBr_2_ and Na_2_SO_3_ system (Table 4) [147]. A broad scope of substrates were efficiently transformed into the desired products, with exclusive formation of the α-bromoketone observed. Nonetheless, the long reaction times and the use of the non-commercially available TsNBr_2_ could be a major hindrance for its widespread application. He and co-workers investigated the same reaction using a commercially available halogenating reagent, 1,3-dibromo-5,5-dimethyldantoin (DBDMH) (Table 4) [157]. The authors illustrated this route’s versatility as it yielded α-bromo-, α-chloro- and α-iodoketones without the need to alter the reaction conditions (this was achieved simply by changing the halogenating reagent, 1,3-dihalo-5,5-dimethyldantoin).

Pitchumani and others established a route involving the addition of Br_2_ to phenylacetylene in cyclodextrin (CD) [158]. An alkyne-CD complex was formed upon the interaction of the substrate in the cavity of the cyclodextrin molecule. The experimental data showed that formation of 2-bromo-1-phenylethanone is enhanced with increased cavity size. In α-CD (6 glucose units), only 13% of the ketone was observed, whereas in γ-CD (8 glucose units), the GC yield of the ketone increased to 46%. This route stands out as it is the first to demonstrate such a complexation effect on the selectivity of oxyhalogenation reaction.

**Scheme 61:**
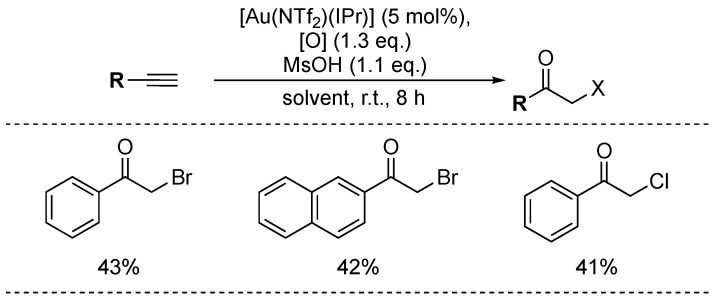
[Au(NTf_2_)(IPr)] catalysed oxyhalogenation.

A gold-based oxidative halogenation method was designed by Xiang and Zhang using [Au(NTf_2_)(IPr)] as a catalyst, 8-methylquinoline *N*-oxide as the oxidant and methanesulfonic acid (MsOH) [159]. One α-chloroketone and two α-bromoketones were obtained via the halide abstraction of the solvent (i.e., dichloro- or dibromoethane). The results, illustrated in Scheme 61, show the poor catalytic activity of the selected Au^I^ complex in this transformation.

**Scheme 62:**
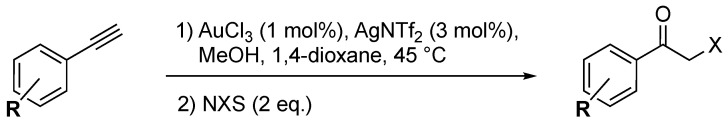
Au^III^-catalysed oxyhalogenation of terminal alkynes.

In 2017, Xing and co-workers demonstrated a Au^III^ system accommodating the oxyhalogenation of terminal alkynes (Scheme 62) [160]. This route yielded aromatic, aliphatic and heteroaromatic α-bromoketones (15 in total) in good to excellent yields (73–91%). The versatility of this route was further highlighted as it also generated five α-chloroketones in good to excellent yields (75–90%) and two α-iodoketones in good yields (81–85%). Although this route seemed to be promising, it brings a number of limitations as the use of silver salts imposed several drawbacks; their limited air- and light-stability, as well as the known interference of Ag in the Au^I^ catalysis [161]. The proposed mechanism involved the formation of the ketone first, followed by the halogenation when NXS is added. The authors proposed that the formation of the ketone is initially driven by the Au-catalysed addition of MeOH to the alkyne; however, in our opinion, this would be improbable. The more plausible route would be the Au-catalysed hydration of the alkyne, most likely using residual water present in the solvents used.

## 4. Oxidative Halogenation of Secondary Alcohols

**Scheme 63:**
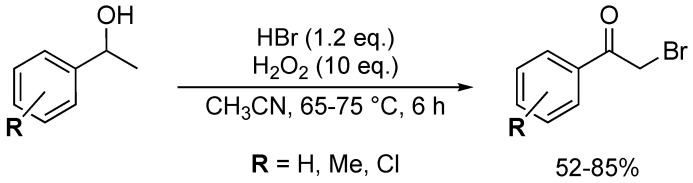
H_2_O_2_/HBr system for the oxidation of secondary benzylic alcohols.

Closely related to the typical oxyhalogenation route, this method relies on the prerequisite setup of a secondary alcohol group and offers straightforward access to α-haloketones. Terent’ev and co-workers developed a H_2_O_2_/HBr system which converted three secondary benzylic alcohols into their corresponding α-bromoketones in moderate to good yields (52–85%) (Scheme 63) [162]. The system was not compatible with *ortho*-substituted 1-phenylethanol derivatives, as the reaction yield dropped significantly (52%). The developed method showed outstanding selectivity, as exclusive formation of the α-haloketone was observed. The authors claimed that the role of the ratio HBr/H_2_O_2_ (1.2:10) is crucial to obtain these results. H_2_O_2_ is most likely responsible for oxidizing the corresponding alcohol to generate the carbonyl moiety and for oxidizing Br^−^ to Br_2_ (or HOBr), the active bromination agent.

**Scheme 64:**
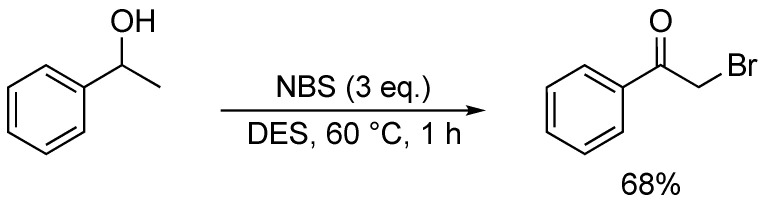
Urea-choline chloride-based deep eutectic solvent (DES) in the oxidative bromination of secondary alcohols.

The dual role of a urea-choline chloride-based deep eutectic solvent (DES) in the oxidative bromination of secondary alcohols was demonstrated by Azizi and co-workers (Scheme 64) [163]. 2-Bromo-1-phenylethanone was obtained in 68% yield when adding three equivalents of NBS to the corresponding alcohol in the DES. Remarkably, the DES both acted as a catalyst system and solvent in this transformation. Recent studies illustrated the superiority of DES in comparison to ILs, linked to their non-toxic nature, easy and low-cost synthesis [164]. Although the mechanism was not fully disclosed, NBS was most likely responsible for both the bromination and oxidation, with DES activating the N-Br bond.

**Scheme 65:**
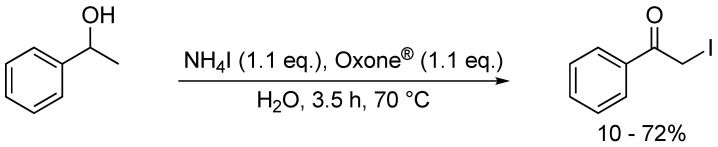
Synthesis of iodomethyl ketones from secondary alcohols.

Narender and co-workers established an aqueous, green and efficient way to generate α-iodoketones from secondary alcohols via addition of NH_4_I and Oxone^®^ (Scheme 65) [165]. The procedure generated a yield of 72% of 2-iodo-1-phenylethanone, but the yield dropped significantly for alcohols bearing substituted aromatic rings (i.e., *p*-Me, *p*-Br, *p*-NO_2_, *m*-OMe). The developed method proved inefficient for substituted 1-phenylethanol derivatives. In 2017, the authors updated the procedure in order to access α-bromoketones via their corresponding alcohols upon the addition of NH_4_Br (2 equiv.) and Oxone^®^ (3 equiv.) [166]. This altered procedure yielded 10 α-bromoketones in low to excellent yields (38–94%). Nitro-substituted benzylalcohols appeared to be deactivated in the developed system. Furthermore, undesired ring bromination exclusively occurred for methoxy substituted 1-phenylethanol derivatives. Despite the progress made in terms of sustainability, this route still shows a rather moderate oxyhalogenation efficiency and selectivity.

**Scheme 66:**
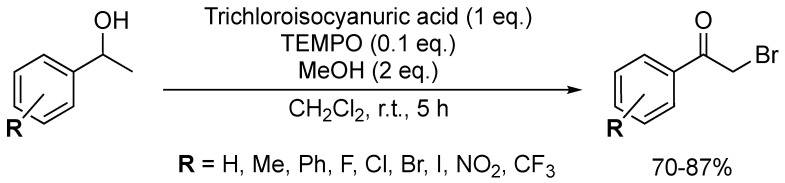
Oxychlorination of 1-phenyl-ethanol by Studer and co-workers.

In 2014, a method for the oxychlorination of 1-phenyl-ethanol was reported by Studer and co-workers (Scheme 66) [167]. Ten α-chloroketones were isolated in good to excellent yields (70–87%) by stirring the alcohol substrates with a mixture of trichloroisocyanuric acid (1 equiv.), TEMPO (0.1 equiv.) and MeOH (2 equiv.) in DCM for 5 h. The presence of electron-donating and -withdrawing groups on the substrates was well tolerated. The protocol stands out as it employs a commonly used bleaching and disinfectant agent (i.e., trichloroisocyanuric acid) in a chemical process.

## 5. Oxidation of Functionalised Hydrocarbons

A different approach to circumvent the problems associated with the direct halogenation route involved using functionalised hydrocarbons as synthons for α-haloketones. In these cases, the halogen is already affixed to the substrate, thus simplifying the process. However, in some of these cases, the substrates could be found as intermediates in the above-mentioned processes (i.e., direct halogenation and oxyhalogenation). Additionally, overall, the fixation of the halogens on the substrates employed in this section could also be burdensome and in some cases limited.

### 5.1. Haloalkanes as Substrates

**Scheme 67:**
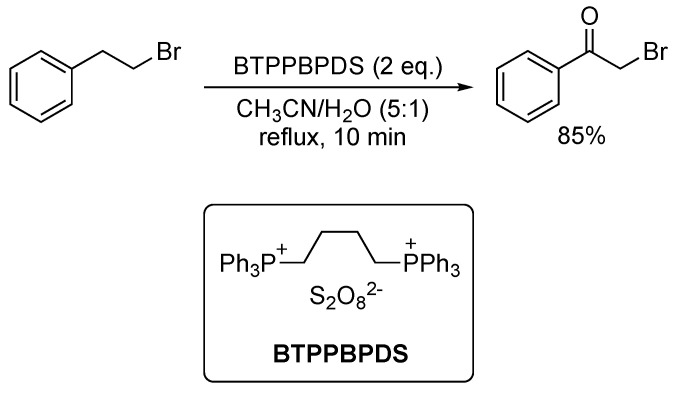
Oxidation of (2-bromoethyl)benzene into the corresponding bromoketone.

Mohammadi and co-workers described the synthesis of 2-bromo-1-phenylethanone from its corresponding bromoalkane in 85% yield [168]. The unconventional 1,4-bis(triphenyl phosphonium)butane peroxodisulfate was employed as the oxidant (Scheme 67). The reaction time was quite short; however, only one example was shown.

Similarly, Jiang and co-workers established a photocatalytic oxidation procedure for the oxidation of a broad range of benzylic alkanes [169]. The corresponding carbonyl compounds were obtained upon the addition of a dicyanopyrazine-derived chromophore (5 mol%), *N*-hydroxyimide (0.2 equiv.) and FeC_2_O_4_·2H_2_O (0.1 equiv.), with subsequent irradiation of the obtained mixture using a 3 W blue LED light. The authors showed only two examples of α-bromoketones, i.e., 2-bromo-1-phenyl-ethanone and its *para*-chloro-substituted derivative, in 83% and 55% yields, respectively. The extremely long reaction times (63–66 h) were also quite limiting.

### 5.2. Haloalkenes as Substrates

**Scheme 68:**
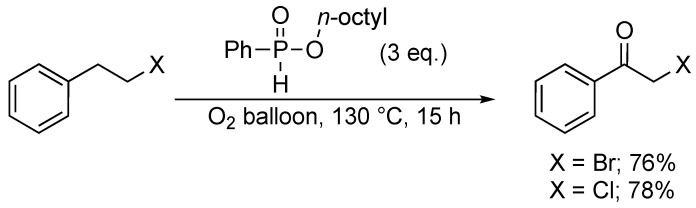
*n*-Octyl phenylphosphinate-mediated oxidation of benzyl haloalkanes.

Synthesis of 2-chloro- and 2-bromo-1-phenylethanone in, respectively, 78 and 76% yield using molecular oxygen as oxidant was described by He and co-workers (Scheme 68) [170]. The developed method involved stirring a mixture of *n*-octyl phenylphosphinate (3 equiv.) and the haloalkene at 130 °C for 15 h in the presence of a O_2_ balloon. This route generated only one side product, i.e., water, highlighting its sustainability.

**Scheme 69:**
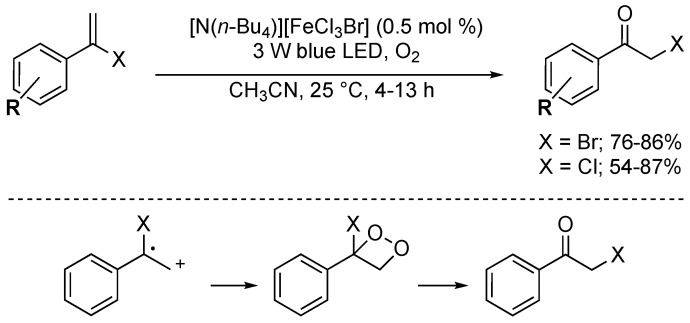
Photocatalytic oxidation of vinyl halides.

Jiang and co-workers developed a photocatalytic oxidation of vinyl halides [171]. Five α-bromoketones and five α-chloroketones were obtained in moderate to excellent yields (Scheme 69). The presence of electron-withdrawing (e.g., F, NO_2_) and -donating (e.g., Me) substituents on the aromatic ring was well tolerated by the catalytic system. The mechanism involved a Fe(III)-Fe(II)-Fe(III) catalytic cycle for the O_2_-assisted radical oxidation of the vinylic substrates followed by the halogen rearrangement of the terminal position.

An alternative Fe^III^ catalytic oxidation protocol was designed by Xiao [172]. Two different methods were reported as illustrated in Table 5. Method 1 exclusively generated the desired α-bromoketone, while the second method yielded de-halogenated ketones as important side products for some substrates. The ligand’s properties thus seemed to have a considerable effect on the protocol selectivity. Nonetheless, a detailed comparison between both methods is difficult due to the limited scope of method 1.

### 5.3. Haloalcohols as Substrates

Haloalcohols or halohydrins were also intermediates in the oxyhalogenation reactions discussed above. Therefore, it is important to note the close relationship between the two sections and that protocols discussed in this section could also help to advance the oxyhalogenation strategies.

Wang and Dong independently developed a micellar catalytic oxidation of alcohols [173,174]. Both authors reported the successful conversion of 2-bromo-1-phenylethanol into its corresponding ketone (Table 6). Wang and co-workers enhanced the substrate’s water solubility upon addition of CTAB, a commercially available cationic surfactant, to the reaction medium. Similarly, Dong and co-workers ensured the sufficient solubility of the substrate by adding GMPGS-2000, a non-ionic surfactant. Both procedures exploited the concept of micellar catalysis, which truly distinguished them from the state-of-the-art. However, one major flaw of these micellar catalytic systems concerns the often tedious work-up, attributed to the difficult separation of the organic and aqueous phase [175].

**Scheme 70:**
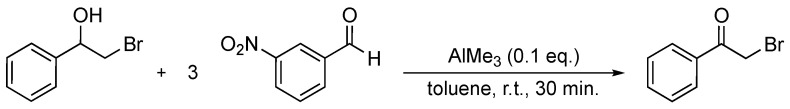
AlMe_3_-catalysed Oppenauer oxidation.

A copper-catalysed oxidation of secondary alcohols was designed by Liu and co-workers [176]. 2-Bromo-1-phenylethanone was isolated in 95% yield upon addition of Cu(OTf) (0.05 equiv.), TEMPO (0.05 equiv.), (*S*)-5-(pyrrolidin-2-yl)-1*H*-tetrazole (0.05 equiv.) and KO*t*Bu (1 equiv.) to the corresponding alcohol in dimethylformamide (DMF) at room temperature. However, no reaction/substrate scope was disclosed. Another limited protocol was developed by Nguyen and co-workers using AlMe_3_-catalysed Oppenauer oxidation (Scheme 70) [177]. The scope again consisted of one α-haloketone, i.e., 2-bromo-1-phenylethanone, making it difficult to assess the method’s efficiency.

**Scheme 71:**
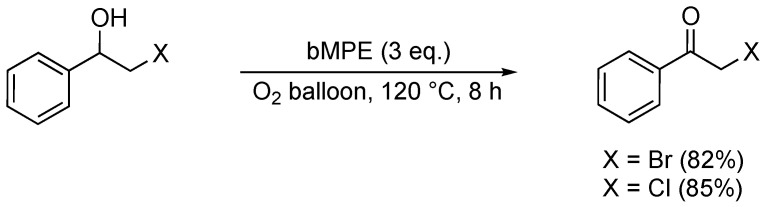
Solvent-free oxidation of functionalised benzylalcohols.

He and co-workers described the bis(methoxypropyl)ether (bMPE)-promoted oxidation of benzylic alcohols [178]. The procedure yielded one α-bromo- and one α-chloroketone in good yields (Scheme 71). Although again the scope was limited, the main advantage of this protocol is its solvent-free conditions.

In 2014, Gotor published a *T. versicolor* Lacasse and TEMPO system, which converted haloalcohols into their corresponding ketones [179]. *T. versicolor* Lacasse is an extracellular enzyme excreted by white and brown fungi. A broad scope of α,α-dihalogenated aryl ketones were obtained in good to excellent yields. The chemoenzymatic procedure also yielded one α-halocarbonyl compound, i.e., 2-chloro-1-phenylethanone, at a yield of 62%. Despite the mild reaction conditions, the long reaction times (24 h) and the moderate yield still limited the method’s applicability.

## 6. Hydration of 1-Haloalkynes

The hydration of haloalkynes is a less commonly described approach to access α-haloketones. Despite the plethora of work that involved the hydration of (terminal) alkynes [180,181,182,183,184,185,186,187,188], this strategy has only scarcely been employed in the synthesis of α-haloketones. The hydration is an excellent example of ideal atom economy. Furthermore, this transformation often only requires the presence of one reagent, i.e., H_2_O, highlighting its green character. This route thus offers immense value from both economic and environmental perspectives.

### 6.1. Metal-Catalysed Hydrations

**Scheme 72:**
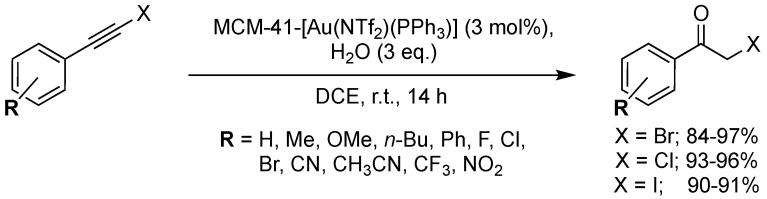
The Au^I^ hydration reaction by Cai and co-workers.

Similarly to the hydration of alkynes [187,188], gold complexes have exhibited an excellent catalytic activity in the hydration of 1-haloalkynes [189]. Cai and co-workers described the hydration of 1-haloalkynes catalysed by a gold catalyst immobilised on mesoporous MCM-41 resin [190]. The versatility of this heterogeneous catalytic route was demonstrated by its broad substrate scope. Sixteen α-bromo, four α-chloro and four α-iodoketones were obtained in excellent yields (Scheme 72). The authors claimed that the catalyst can be recycled and reused for eight consecutive runs without significant loss of catalytic activity. Additionally, they stated that after these eight runs, gold leaching did not occur. The excellent atom economy, mild reaction conditions, the generality and catalyst recyclability elevated the importance of this protocol compared to the state-of-the-art. In 2019, the same group reported an updated procedure relying on the in situ activation of MCM-41-[Au(Cl)(PPh_3_)] (2 mol%) by AgNTf_2_ [191]. Despite the decreased catalyst loading, this route’s scope was very limited, as only 2-bromo-1-phenyl-ethanone (92%) was obtained.

**Scheme 73:**
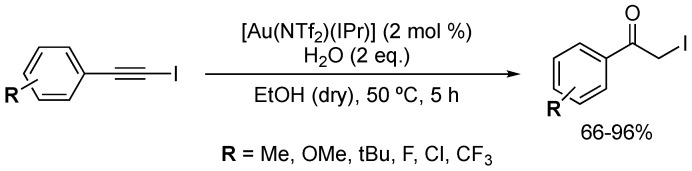
The Au^I^ hydration iodoalkynes by Cazin and co-workers.

Another gold-catalysed protocol using [Au(NTf_2_)(XPhos)] to perform the hydration of 1-bromo and 1-chloroalkynes was designed by He and Xiang [192]. Ten aromatic α-bromoketones and two aromatic α-chloroketones were isolated in low to excellent yields (up to 97% and 91%, respectively), upon of addition of 3 mol% of the catalyst and water (3 equiv.) to the 1-haloalkyne in dichloroethane. The presence of electron-donating (i.e., Me, *t*-Bu, OMe) and -withdrawing (i.e., F, Br) groups on the substrates was well tolerated. However, the hydration efficacy of the system decreased significantly for sterically hindered substrates. The hydration of the *ortho*-substituted 1-haloalkynes yielded the corresponding ketones in trace amounts. Furthermore, the protocol demonstrated a substrate specific nature, as it did not accommodate the hydration of 1-iodoalkynes. More recently, Cazin and co-workers developed an efficient catalytic protocol for the Au(I)-catalysed hydration of 1-iodoalkynes (Scheme 73) [193]. The use of Au(I)-NHC catalysts enabled the straightforward synthesis of several aromatic α-iodomethyl ketones in good to excellent yields. The utility of this simple method was further highlighted by showcasing iodination/hydration and hydration/oxidation sequential protocols leading to the construction of molecular complexity.

A broad scope of α-haloketones was also obtained via the Cu(OAc)_2_·H_2_O and trifluoroacetic acid (TFA) promoted hydration system developed by He (Table 7) [194]. This route coped well with substrates bearing electron-withdrawing and -donating groups. The authors claimed that the catalyst can be recycled and reused for five consecutive runs without loss in hydration activity. In 2014, Lui and co-workers reported the remarkable catalytic activity of the AgF/TFA system in the hydration of alkynes (Table 7) [195]. This system tolerated a wide variety of functional groups attached to the phenyl ring (i.e., CF_3_, Br, Me, NO_2_, OMe, etc.). The procedure showed a substrate specific character as it did not mediate the hydration of 1-iodoalkynes. The use of the strongly acidic TFA is a major drawback, and negatively impacts the functional group tolerance of both routes.

**Scheme 74:**
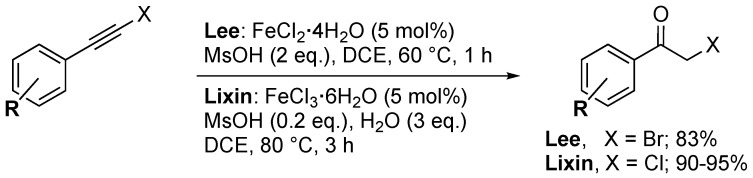
Iron chloride-catalysed hydration of 1-bromo and 1-chloroalkynes.

Lee and Lixin independently described an FeCl_x_·yH_2_O-catalysed hydration [196,197]. Their systems required the presence of methanesulfonic acid (MsOH) in order to transform 1-haloalkynes in sufficiently high yields (Scheme 74). The substrate scope was rather limited, as Lee’s procedure generated one α-bromoketone and Lixin’s reported only three aromatic α-chloroketones. The acidic nature of these protocols further narrowed the hydration scope, as acid-sensitive functional groups (e.g., OH, NH_x_) did not tolerate these conditions.

**Scheme 75:**
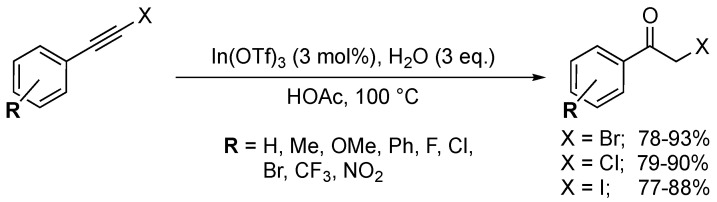
In(OTf)_3_-catalysed hydration of 1-bromoalkynes by Xiao and co-workers.

Xiao and co-workers designed an In(OTf)_3_ (10 mol%)-catalysed hydration optimised for 1-bromoalkynes (Scheme 75) [198]. The hydration reaction was only effective if carried out in an acidic solvent, i.e., HOAc. Together with the high reaction temperature, this route requires harsh hydration conditions, which limits its applicability. Nevertheless, 16 α-bromo, 3 α-chloro and 3 α-iodoketones were obtained in good to excellent yields (78–93%). Despite the acidic solvent, the system mediated the hydration of an OH-substituted 1-bromoalkyne, affording the corresponding ketone in 82% yield.

**Scheme 76:**
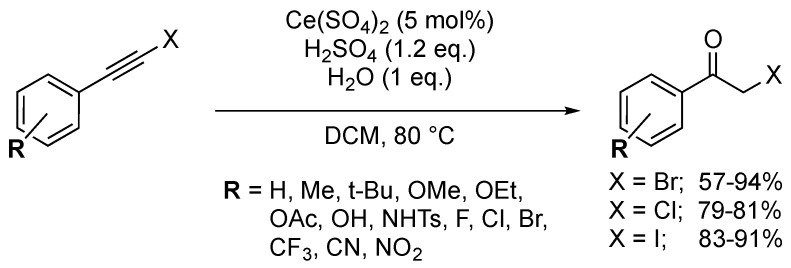
Ce(SO_4_)_2_-catalysed hydration of 1-bromoalkynes by Xiao and co-workers.

Recently, Xiang and co-workers designed a highly versatile Ce(SO_4_)_2_-catalysed hydration [199]. The addition of sulfuric acid to the 1-haloalkyne with 5 mol% of Ce(SO_4_)_2_ in DCM, generated 30 α-haloketones in moderate to excellent yields (Scheme 76). Despite the acidic nature of the protocol, acid-sensitive groups (i.e., OH and NHTs) were well tolerated. The presence of steric bulk close to the triple bond appeared to have a considerable effect on the hydration efficiency.

### 6.2. Non-Metal-Catalysed Hydrations

**Scheme 77:**
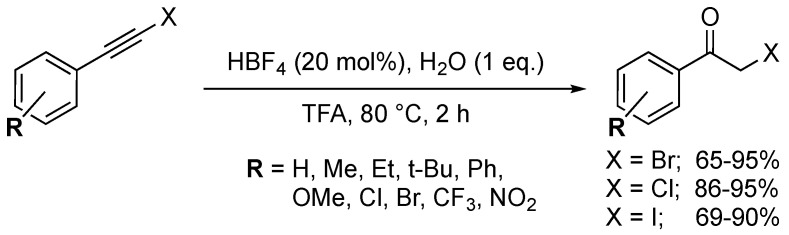
HBF_4_-catalysed hydration of 1-bromoalkynes by Wang and co-workers.

In 2016, a metal-free hydration of 1-haloalkynes in 2,2,2-trifluoroethanol was described by Wang and co-workers (Scheme 77) [200]. Thirteen α-bromo, seven α-chloro and eight α-iodoketones were obtained in good to excellent yields. Nonetheless, *ortho*-substituted 1-haloalkynes were less efficiently hydrated due to the presence of steric bulk close to the alkyne centre.

## 7. Miscellaneous Routes

### 7.1. α-Functionalised Ketones as Substrates

**Scheme 78:**
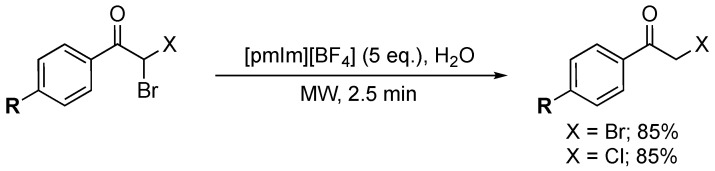
Selective debromination of α,α-dihaloketones.

In 2018, Baire published the selective monodehalogenation of α,α-dihaloketones [201]. 2-Iodo-1-phenylethanone was isolated in 64% yield upon stirring the α,α-diiodo-product for 10 h in a CH_3_CN/H_2_O mixture. The procedure yielded an inseparable mixture of the α-haloketone and α,α-dihaloketone for 2-bromo-1-phenylethanone. Alternatively, Ranu et al. established an IL-mediated selective debromination method in which 1-methyl-3-pentylimidazolium tetra-fluoroborate ([pmIm]BF_4_) acted as both catalyst and reaction medium [202]. Two aromatic α-bromoketones (R = H, Me) and one α-chloroketone (R = Me) were generated in good yields (Scheme 78). This route exhibited short reaction times, excellent efficacy and selectivity, and the use of a recyclable and readily available catalyst.

**Scheme 79:**
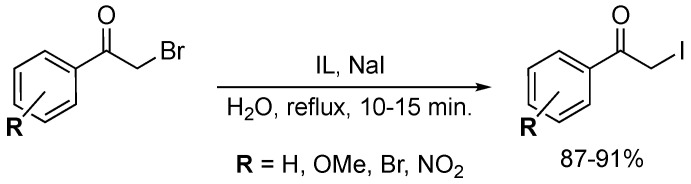
Access to α-iodoketones using a poly(ionicliquid)/NaI system.

In the last decade, Sayyahi and co-workers dedicated considerable effort towards the development of an effective S_N_2 substitution protocol yielding α-functionalised ketones from their corresponding α-bromoketones [203,204,205,206]. Their most recent work involved an imidazolium poly(ionic liquid)-based substitution (Scheme 79). Four aromatic α-iodoketones were obtained in excellent yields (87–91%) upon the addition of the IL and NaI to the substrate in H_2_O at 60 °C. The authors claimed that the IL can be recycled and reused in up to five consecutive runs without significant loss in activity. This route has some promising features, i.e., catalyst recycling, aqueous solvent and mild reaction conditions; however, it still suffers from the need to synthesise the α-bromoketone substrate.

**Scheme 80:**
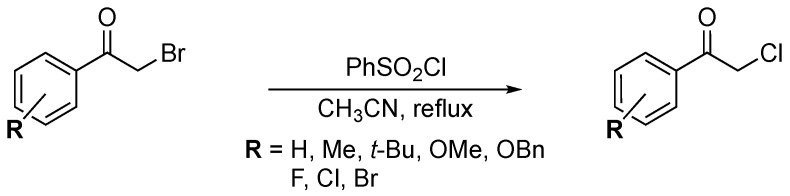
PhSO_2_Cl-mediated substitution of α-bromoketones.

An alternative substitution procedure, developed by Zhang and co-workers, generated 14 α-chloroketones in quantitative yields from α-bromoketones (Scheme 80) [207]. This route tolerated the presence of electron-donating and -withdrawing aromatic substituents. Additionally, sterically hindered substrates were tolerated. This procedure afforded quantitative yields, thus avoiding chromatographic purification of the products.

**Scheme 81:**
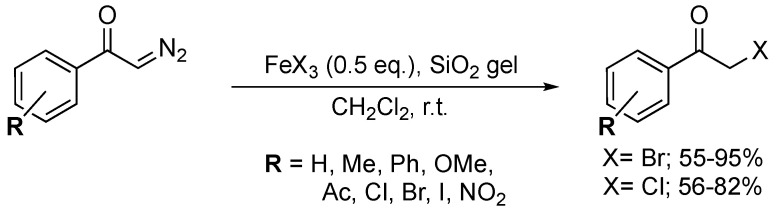
Halogenation of α-diazoketones by Wang and co-workers.

Wang and co-workers designed a synthetic protocol converting α-diazoketones in α-halo-ketones (Scheme 81) [208]. The protocol yielded ten α-chloroaryl ketones in 56–82% when stirring a mixture of FeCl_3_ (0.5 equiv.), silica gel (100 mg) and the diazo compound in DCM for 10 min. Furthermore, seven α-bromoaryl ketones were obtained in moderate to excellent yields (55–95%) by changing the Lewis acid to FeBr_3_. This route’s versatility was demonstrated through its tolerance towards electron-donating (e.g., Me, OMe) and -withdrawing (e.g., I, Cl, NO_2_) aromatic substituents, as well as sterically hindered substrates.

**Scheme 82:**
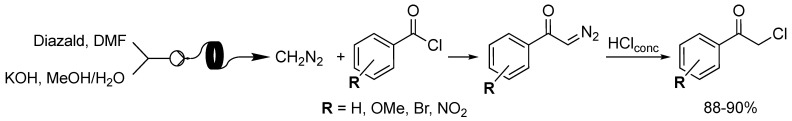
Continuous process for the synthesis of α-chloroketones.

Kappe and Gutmann developed a continuous process for the synthesis of α-chloroketones from their acetyl chloride derivatives (Scheme 82) [209,210]. The protocol involved mixing a *N*-methyl-*N*-nitroso-*p*-toluenesulfonamide (diazald) solution in DMF and a KOH solution in MeOH/H_2_O with the starting material to afford the α-diazoketone intermediate. Subsequent addition of HCl allowed the isolation of four aromatic α-chloroketones in excellent yields (88–90%). The on-demand generation and direct consumption of diazomethane or its precursor certainly eliminate human exposure to the reagent, thus reducing the risk of explosive decomposition.

**Scheme 83:**
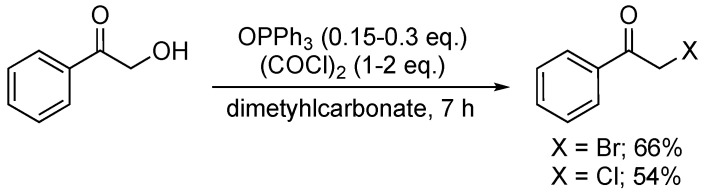
Appel halogenation of α-hydroxy ketone.

Jordan and co-workers aimed to establish a sustainable procedure for the Appel halogenation, which converts alcohols in alkyl halides [211]. Although a broad scope of halides was obtained, the procedure only yielded two aryl α-haloketones (Scheme 83). The authors claimed that O=PPh_3_ was a recyclable organocatalyst; however, tedious procedures are required to completely remove the oxide from the reaction mixture [212]. Lai and co-workers described an alternative procedure which also accessed 2-choro-1-phenylethanone from its α-hydroxy ketone [213]. Stirring a cooled mixture of the ketol, tosyl chloride (1.1 equiv.), triethylamine (2 equiv.) and 4-dimethylaminopyridine (2 mol%) in DCM yielded the α-chloroketone in 83% yield. This procedure seemed to exhibit a higher efficacy than the previous one; however, its scope was still limited and did not permit a thorough comparison with other routes.

**Scheme 84:**
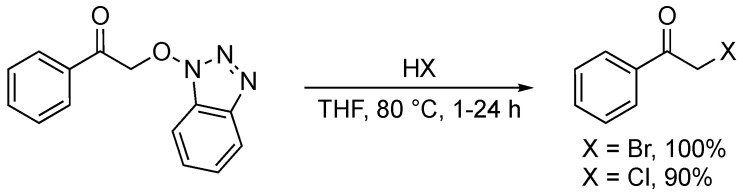
α-Haloketones accessed via α-hydroxybenzotriazole functionalised ketones.

Zhang and Tang designed an S_N_2 substitution method yielding α-haloketones from α-hydroxy-benzotriazole functionalised ketones (Scheme 84) [214]. Their method is quite practical; however, it relies on the prior placement of an hydroxybenzotriazole group on the starting substrate which is not entirely straightforward, thus limiting the scope significantly.

**Scheme 85:**
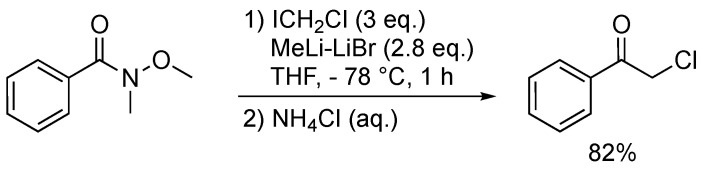
ICH_2_Cl- and MeLi-mediated synthesis of 2-chloro-1-phenylethanone.

An alternative procedure describes the synthesis of 2-chloro-1-phenylethanone from the phenyl Weinreb amide (Scheme 85) [215]. The harsh reaction conditions and the use of unstable reagents (i.e., ICH_2_Cl) limit the protocol’s application considerably.

### 7.2. Alternative Substrates

**Scheme 86:**
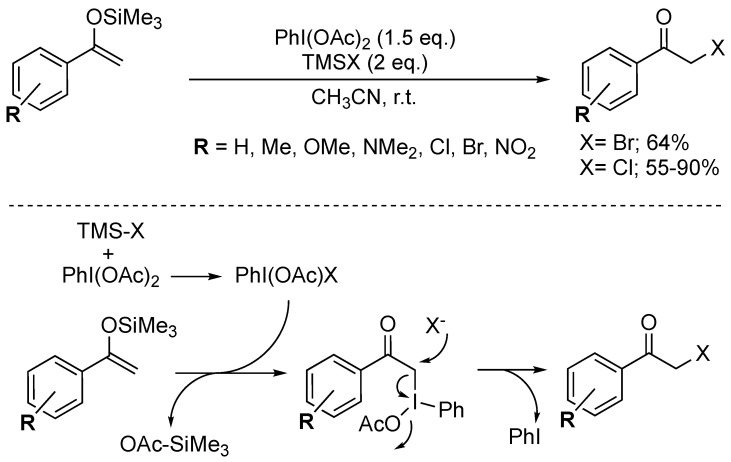
Silyl enol ethers as suitable synthons for aromatic α-haloketones.

Yingpeng, Yulai and co-workers demonstrated that silyl enol ethers are suitable synthons for aromatic α-haloketones (Scheme 86) [216]. One α-bromo- and 12 α-chloroketones were generated upon the addition of (diacetoxyiodo)benzene (1.5 equiv.) and trimethylsilyl halide (2 equiv.) to the silyl enol ether in acetonitrile. 2-Bromo-1-phenylethanone was isolated in 64% yield and a set of α-chloroketones were obtained in moderate to excellent yields (55–90%). The procedure tolerated the presence of electron-donating (e.g., Me, OMe, NMe_2_) and -withdrawing (e.g., Cl, Br) substituents on its aromatic substrates. The mechanism as described in Scheme 86 highlighted the initial formation of a ketone intermediate bearing an iodo-leaving group, followed by nucleophilic substitution with the corresponding halide to afford the desired compound.

**Scheme 87:**
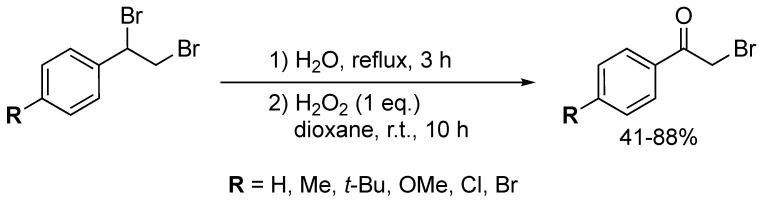
Synthesis of α-bromoketones via *vic*-1,2-dibromides.

Patil, Adimurthy and Ranu established the synthesis of α-bromoketones from their corresponding *vic*-1,2-dibromides [217]. As shown in Scheme 87, six haloketones were isolated in moderate to excellent yields. Selectivity issues occurred for the OMe-substituted dibromides, as the brominated arylhydrin was obtained as an important side product. This problem and the relatively long reaction times are this route’s main disadvantages.

**Scheme 88:**
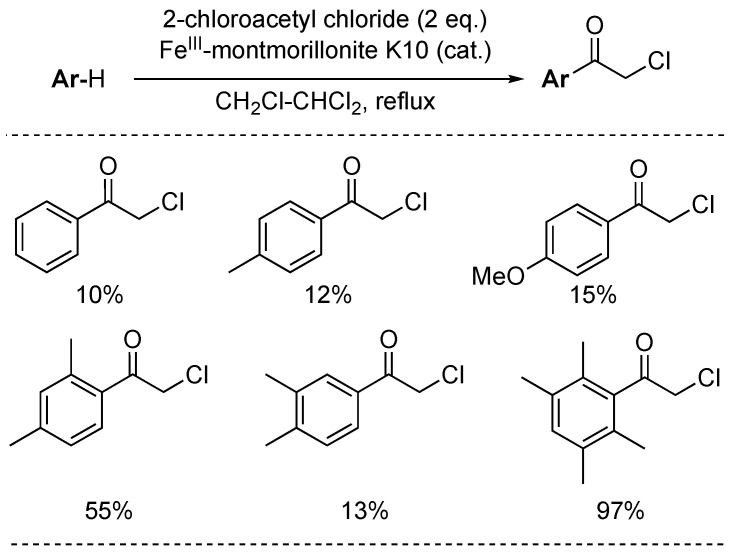
Chloroacetylation of substituted arenes.

α-Chloroketones were also accessed via the chloroacetylation of arenes (Scheme 88) [218]. This Fe^III^-catalysed procedure exhibits a low efficacy; however, when highly sterically hindered arenes were used, a remarkable increase in yield was observed.

Ram and Manoj designed the Cu^I^ promoted synthesis of α-haloketones from trichloromethyl carbinols [219]. Fifteen carbinols were efficiently converted upon addition of two equivalents of CuCl and bipyridine in dichloroethane. Subsequent stirring for 3 h under reflux yielded the corresponding α-chloroketones in 82–96%. This route coped well with the presence of electron-withdrawing (e.g., Cl, Br, NO_2_) and -donating (e.g., Me, OMe) groups on substrates.

**Scheme 89:**
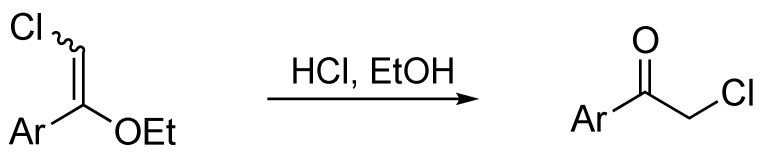
Yoshimatsu’s oxidation protocol for enolethers.

In 2007, Yoshimatsu et al. reported an HCl/EtOH-based system yielding 15 (hetero)aromatic α-chloroketones from their enol ethers in 18–100% (Scheme 89) [220], though it should be noted that harsh reaction conditions are required to access the enol ether compounds.

**Figure 15:**
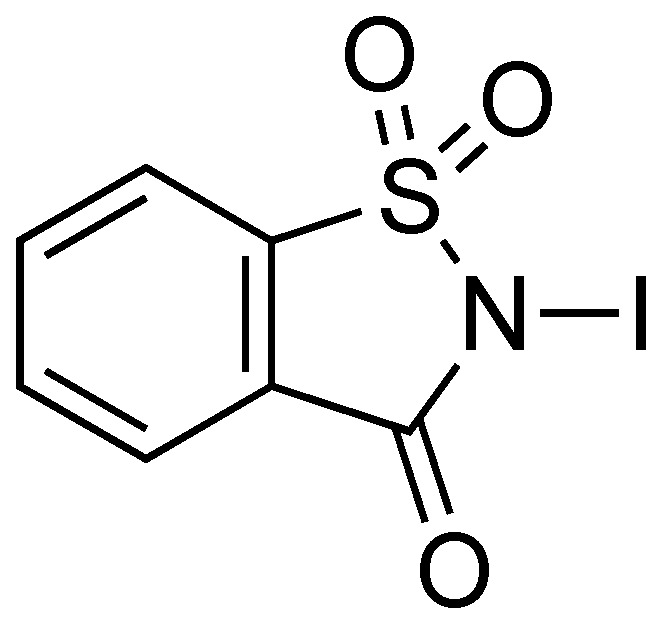
Structure of *N*-iodosaccharin.

Enolacetates have also proven to be suitable synthons for α-haloketones [221]. Electron-poor, electron-rich and sterically hindered enolacetates were efficiently transformed upon the addition of *N*-iodosaccharin (Figure 15). Despite the broad scope, only one aromatic α-iodoketone (i.e., 2-iodo-1-phenylethanone) was isolated in 56% yield.

## 8. Conclusions and Outlook

Overall, this review analysed several methodologies to access aromatic α-haloketones. In the last two decades, a plethora of halogenation agents have been developed, though X_2_ remains the most abundantly used. Alternative and more sustainable halogen sources are beginning to have a significant impact in the literature, as demonstrated by the overview given herein. *N*-halosuccinimides, HX and MX strategies appear to be the most promising, as they showcase a better balance between reactivity and associated hazards. Ionic liquid-based halogenation agents could also be a step forward in the right direction, though it is still too early to properly assess these protocols, since their use is still limited due to their cost and restricted (commercial) availability. It is our hope that the combination of the current knowledge, collated in this review, will undoubtedly lead to improved strategies soon.

The community has strived to tackle the direct halogenation issues. Several oxyhalogenation methods for alkenes, alkynes and secondary alcohols have been described in the last 20 years. However, these only partially addressed the creation of a general and useful method, as some methods require the presence of an acidic promotor or have demonstrated efficacy and/or selectivity limitations. On the other hand, research showed that the oxidation of functionalised hydrocarbons could very well yield the synthetically valuable α-haloketones. This route’s value in organic synthesis is still limited due its moderate activity, selectivity and/or versatility. Additionally, the use of non-readily (commercially) available starting materials further hinders their applicability.

In comparison to most protocols dedicated to the direct halogenation of ketones, the hydration of 1-haloalkynes has only scarcely been explored in the last two decades. The latter route can be considered as the best alternative for the direct halogenation, especially considering its high atom efficiency and the use of only water as reagent.

The current field still possesses a remarkable growth potential, and we therefore hope to see more advances soon that directly address the numerous problems highlighted in this review.

## Figures and Tables

**Table 1:** The bromination scope reported by Krupadanam.

R	R′	R″	Yield (%)	
H	H	H	85	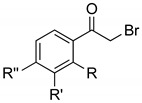
H	H	Cl	70	
Cl	H	Cl	60	
H	NO_2_	H	70	
H	H	Br	75	
H	H	F	85	
H	H	OMe	70	
H	H	OCH_2_Ph	65	

**Table 2:** Aqueous H_2_O_2_-based oxyhalogenation methods.

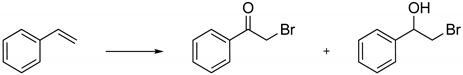			
Reaction Conditions	α-Bromoketone (%)	Bromohydrin (%)	Ref.
HBr (2 equiv.), H_2_O_2_ (1 equiv.), H_2_O, r.t.	12	53	[141]
NBS (2 equiv.), H_2_O, r.t.	19	81	[141]
30% aq. HBr (1.7 equiv.), H_2_O_2_ (3 equiv.), H_2_O, r.t.	94	4	[140]

**Table 3:** Photo-oxidative synthesis of phenylacylhalides.

X	Reaction Conditions	Scope (Yield)	Aromatic Substituents
I	I_2_ (0.6 equiv.), *hv*, H_2_O/EtOAc	7 α-iodoketones (43–88%)	H, *t*-Bu, Me, OMe, Cl, Ph
Br	48% aq. HBr (1.1 equiv.), *hv*, H_2_O/EtOAc	7 α-bromoketones (trace–86%)	H, *t*-Bu, Me, OMe, Cl, Ph

**Table 4:** Phukan and He’s oxyhalogenation procedures.

Reaction Conditions	Scope (Yield)	Aromatic Substituents	Refs.
TsNBr_2_ (2 equiv.), Na_2_SO_3_ (8 equiv.), EtOAc/acetone/H_2_O, r.t, 24 h	17 α-bromoketones (75–80%)	H, Me, *t*-Bu, *n*-Pr, F, Br, Cl	[147]
DBDMH (1.2 equiv.), ethylene thiourea (0.15 equiv.), acetone/H_2_O, 45 °C	17 α-bromoketones (77–91%) 1 α-chloroketone (78%) 1 α-iodoketones (69%)	H, Me, OMe, OBn, SMe, F, Cl, Br, I, CF_3_	[157]

**Table 5:** Fe^III^-catalysed oxidation of haloalkenes.

Method	Reaction Conditions	Scope (Yield)	Aromatic Substituents
**1**	Fe(OTf)_3_ (1.15 mol%), L4 (1.15 mol%), DCE, 75 °C, 8 h	2 α-Bromoketones (91–92%)	H, Cl
**2**	Fe(OTf)_3_ (1.15 mol%), L1 (1.15 mol%), O_2_ (1 atm), DCE/DBE 75 °C, 8 h	4 α-Bromoketones (65–99%) 2 α-Chloroketone (50–70%) 1 α-Iodoketones (70%)	H, Me, OMe, F, Cl, Br
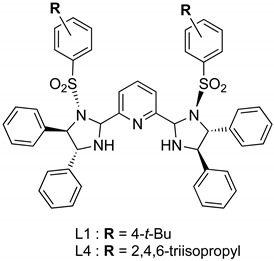			

**Table 6:** Micellar catalytic oxidation of 2-bromo-1-phenylethanol.

Reaction Conditions	Scope (Yield)	Refs.
2-iodosylbenzoic acid (0.01 equiv.), Oxone^®^ (0.525 equiv.), CTAB (3 wt%), H_2_O, r.t., 2 h	2-bromo-1-phenylethanone (95%)	[173]
IBX (1.2 equiv.), GMPGS-2000 (2 wt%), H_2_O, r.t., 24 h	2-bromo-1-phenylethanone (64%)	[174]

**Table 7:** Cu^II^- and Ag^I^-catalysed hydrations.

Route	Catalyst (mol%)	Reaction Conditions	Scope (Yield)
He (2016) [194]	Cu(OAc)_2_·H_2_O (10)	H_2_O (1 equiv.) in TFA at 70 °C for 6 h	15 α-bromoketones (77–96%) 3 α-chloroketones (91–92%) 4 α-iodoketones (90–96%)
Liu (2014) [195]	AgF (5)	H_2_O (1 equiv.) in TFA at 40 °C for 6–16 h	15 α-bromoketones (61–95%) 3 α-chloroketones (81–90%)
